# Novel Antimicrobial-Decorated Polyelectrolytes as Versatile Building Blocks for Multifunctional Hydrogel Nano- and Microparticles

**DOI:** 10.1021/acsomega.5c02518

**Published:** 2025-05-20

**Authors:** Weronika Szczęsna-Górniak, Łukasz Lamch, Alicja Surowiak, Ewa Zboińska, Lilianna Szyk-Warszyńska, Marcin Bartman, Piotr Warszyński, Kazimiera A. Wilk

**Affiliations:** † Department of Engineering and Technology of Chemical Processes, Faculty of Chemistry, 49567 Wrocław University of Science and Technology, Wrocław 50-370, Poland; ‡ Department of Bioorganic Chemistry Faculty of Chemistry, Wrocław University of Science and Technology, Wrocław 50-370, Poland; § Department of Organic and Medicinal Chemistry, Faculty of Chemistry, Wrocław University of Science and Technology, Wrocław 50-370, Poland; ∥ 132074 Jerzy Haber Institute of Catalysis and Surface Chemistry, Polish Academy of Sciences, Kraków 30-239, Poland

## Abstract

The construction of multipurpose particles with functional coatings of varying structure and composition provides the opportunity to modify their physicochemical and biological characteristics. In accordance with the aforementioned, new polyelectrolytes (PEs) decorated with an antimicrobial function (PEs-DAF) were designed and prepared to apply them as building blocks of a various carrier systems. A series of hydrogel nano- and microparticles were developed and coated with an outer PE shell with antimicrobial functionality. To this end, poly­(acrylic acid) (PAA) was grafted with different degrees of substitution (DS) of antimicrobial essential oils such as thymol (THY), menthol (MEN), and carvacrol (CAR) (abbreviated as PAA-X-DS% (X = THY, MEN, CAR; DS = 5,15)) using Steglich esterification under mild reaction conditions. Their structures were confirmed by ^1^H NMR and FTIR spectroscopy. The particles’ morphology and mean diameter were determined by dynamic light scattering (DLS), scanning electron microscopy (SEM), transmission electron microscopy (TEM), and atomic force microscopy (AFM). The physicochemical properties of the novel functional coatings were characterized using quartz crystal microbalance with a dissipation (QCM-D) analysis and spectroscopic ellipsometry. The antimicrobial properties of the functionalized PAA and the alginate microgel particles decorated with these PEs were evaluated against Staphylococcus aureus and Escherichia coli using the agar disc diffusion assay and minimal inhibitory concentration evaluation. The particles exhibited satisfactory antimicrobial activity, and some examples showed higher bioactivity than the functionalized PAAs. Moreover, the designed systems were loaded with resveratrol (RES), a model chemotherapeutic substance, to assess their potential applicability as drug carriers. The analysis proved the effective RES encapsulation and its release in a controlled manner depending on the coating properties. The results found in our study indicate potential therapeutic applications of the new antimicrobial-decorated carrier systems in the treatment of multidrug-resistant pathogenic infections.

## Graphical abstract

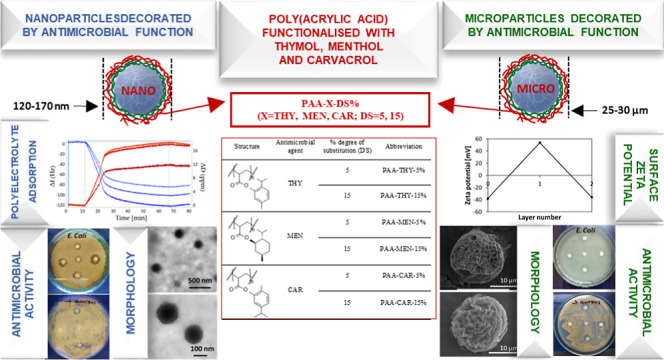

## 1 Introduction

The design of drug delivery systems (DDSs) has attracted considerable interest in the scientific community due to several factors, including improved drug solubility, enhanced chemical and colloidal stability, sustained and prolonged release, and reduced side effects. However, when the drug carriers are administered into an infected area of the body, their exposure to various microorganisms limits the effectiveness of therapeutic treatment.^1^ Thus, protecting carrier systems from bacterial proliferation and pathogenic infection is crucial. One potential solution to this problem is the functionalization of DDSs with various antimicrobial agents, forming multifunctional systems that integrate the key functions of a therapeutic drug and an antibacterial agent.^1^ The incorporation of antimicrobial groups into the carrier surface has been demonstrated to inhibit the growth of bacteria in target cells, thereby reducing side effects and enhancing the efficacy of the treatment.^2^

Modifying the carrier surface represents a promising approach for improving several key properties, including cellular uptake, biodistribution, selective accumulation in tissues, controlled drug release, payload binding capacity, targeted delivery, and prolonged circulation time in the body. This enables the application of these carriers in a wide range of personalized therapies. The functionalization of the carrier particles may be achieved through the utilization of physical and chemical methods, employing a range of functional moieties, including amine, disulfide, thiol, and sulfhydryl groups. Furthermore, incorporating targeting agents, including ligands, antibodies, proteins, antimicrobial peptides, antibiotics, and polymers, can enhance the specificity and efficacy of the carrier particles.^3^ One of the most frequently utilized strategies of carrier surface engineering is the layer-by-layer (LbL) self-assembly technique.^4,5^ It is considered the facile and effective method for obtaining functional coatings on colloidal particles, allowing for the deposition of multilayer structures on the carrier surface through the alternating adsorption of positively and negatively charged polyelectrolytes (PEs) via electrostatic interactions.^6^ The LbL method enables the regulation of coating formation with diverse architectures and functions, employing a spectrum of synthetic and natural polyelectrolyte materials. The characteristics of the carrier system, including biocompatibility, permeability, and chemical and colloidal stability, can be readily modified through the use of LbL shells, thus allowing for the creation of a range of modified carrier systems with specific desired properties. The flexibility and simplicity of the LbL technique render it a widely applied strategy for tailoring PE films’ physicochemical properties. That is accomplished by modifying the deposition parameters, including ionic strength, pH, temperature, and polymer concentration. Consequently, the LbL approach provides the opportunity to design a variety of polymeric carriers with functionalized coatings that control their applicable properties, thereby developing multipurpose systems with the desired features.

A highly efficient strategy for the construction of antimicrobial systems is the modification of carrier surfaces with a variety of functional compounds, including antibiotics, peptides, polymers, or metallic antibacterial agents. Consequently, an intriguing strategy for modifying carriers is the utilization of antimicrobial polymers, which offer several advantages, including minimal residual toxicity, high selectivity, and a prolonged lifetime.^7^ Among the wide range of such polymers, those bearing antibacterial moieties are the most promising for the formation of functional coatings on carrier surfaces.^8^ The antimicrobial groups may be chemically linked with the backbone chain of PE by different labile bonds (e.g., ester, amide, imine or acetate) that are stable at specific pH values.^2^ The stability of these polymeric structures permits the construction of functionalized layers of drug carrier systems with long-lasting antimicrobial activity. Modifying the carrier surface using antibacterial PEs is particularly important due to the growing wave of antimicrobial resistance (AMR) for conventional antibiotics. Worldwide statistics indicate that AMR has become one of the most urgent global health threats of the 21st century. In this context, the development of new pharmaceutical strategies, including alternate antimicrobials, multifunctional delivery systems or combination therapies, is urgently needed to combat multidrug resistance and protect the symbiotic host-microbial balance. Thus, the design and fabrication of a variety of multipurpose DDSs modified with new PEs containing antibacterial moieties as their outer functional coatings may be an excellent, nontraditional and innovative approach to address the trends in drug resistance^2^ effectively. The antimicrobial-decorated carriers may provide: (i) targeted and sustained release of bioactive agents; (ii) longer antibacterial activity in time; (iii) response to specific environmental stimuli, ensuring precise activation of antimicrobial activity; (iv) increased local concentration of antibacterial compounds. In the face of rising AMR, the advanced DDSs functionalized with antimicrobial PEs coatings can offer an efficient, selective, and long-term strategy for combating multidrug-resistant pathogens and safeguarding public health.

The polyelectrolytes decorated with an antimicrobial function (PEs-DAF) can be employed as the fundamental units of various nano- and microparticles. An intriguing category of DDSs are hydrogel carriers, which exhibit an exceptional capacity for water absorption, high swelling potential, tunability, biocompatibility, and biodegradability. It is essential to select an appropriate material for fabricating such carriers.^9^ Natural polyelectrolytes, including sodium alginate (ALG) and chitosan (CHIT), as well as synthetic polyelectrolytes such as poly­(acrylic acid) (PAA), are regarded as excellent building blocks for the fabrication of biocompatible DDSs with payload-controlled release ability. The surface modification of hydrogel particles with PEs-DAF coatings provides the opportunity to develop multipurpose carrier systems that, in addition to their function of encapsulated active compounds, possess additional functionality, such as antimicrobial activity. It enables the formation of multilayered hydrogel particles with desirable applications. Furthermore, functionalizing nano- and microparticles with PEs-DAF layers can prevent bacterial infection, reduce side effects, and improve disease treatment.

The significant benefits of PEs-DAF, when employed as functional coatings, are as follows: (i) the structural diversity and physicochemical properties of PE films can be adjusted; (ii) antimicrobial activity can be controlled by controlling the composition and structure of layers; (iii) the release of encapsulated drugs can be prolonged and controlled; (iv) products can be designed for long-term application. DDSs with antimicrobial functionality have the potential to simultaneously deliver active substances to infected sites while exhibiting long-term antibacterial properties. It is, therefore, imperative that new, advanced PEs-DAF are designed in order to facilitate the functionalization of carrier surfaces and the development of novel, multipurpose polymeric materials.

A literature review reveals that naturally occurring compounds demonstrate highly effective antibacterial activity, particularly in comparison to other antimicrobial agents.^10^ Among the numerous plant-derived substances, essential oils, including thymol (THY), menthol (MEN), and carvacrol (CAR), appear to be promising bioactive agents with low systemic toxicity and significant antimicrobial features. The primary objective of this study was to design and synthesize new PEs decorated with an antimicrobial moiety for their application as building blocks of versatile carrier systems. Thus, we developed, fabricated, and characterized hydrogel nano- and microparticles comprising an ALG core and custom-designed LbL coatings formed by CHIT and PAA functionalized with THY, MEN, or CAR, as the outer antimicrobial layer. Novel PEs-DAF constituted the additional functionality of nano- and microparticles developing multipurpose carrier systems. The use of mild PE and natural-based antimicrobial agents enables the combating of multidrug-resistant bacteria and the avoidance of harmful effects associated with therapy. The synthesis of PAA decorated with THY, MEN, and CAR was conducted using Steglich esterification under mild conditions. The size, morphology and zeta potential of the manufactured particles were examined using scanning electron microscopy (SEM), transmission electron microscopy (TEM), atomic force microscopy (AFM) and dynamic light scattering (DLS). The thickness, mass, and viscoelasticity of the functional films were determined using a quartz crystal microbalance with a dissipation (QCM-D) technique. Furthermore, the impact of the coatings’ type and PE functionalization on the antibacterial properties of formed nano- and microparticles against Gram-positive (Staphylococcus aureus) and Gram-negative (Escherichia coli) bacterial strains was investigated. Additionally, the designed carriers were loaded with resveratrol (RES), a model chemotherapeutic compound, to assess their capacity for drug encapsulation and its release mechanism. The physicochemical and biological properties of the fabricated nano- and microparticles allowed for evaluating their potential applicability as multifunctional DDSs in various antimicrobial and cancer therapies.

## 2 Results and Discussion

The construction of properly designed nano- and microparticles with various functional coatings provides the opportunity to adjust their physicochemical and biological features, thus enabling the development of multipurpose materials for therapeutic applications. Therefore, polyelectrolytes functionalized with different types of naturally occurring antimicrobial agents were employed as building blocks for fabricating a diverse range of nano- and microparticles. The design of multilayered particles coated with an outer layer containing PEs-DAF allowed for the formation of advanced carrier systems that can protect against bacterial proliferation and suppress pathogenic infections, thereby improving disease treatment. The development of multifunctional materials combining several functions, such as the delivery of bioactive substances and the maintenance of antimicrobial properties, promotes treatment efficacy.

### 2.1 Design, Synthesis and Characterization of PAA Decorated by THY, MEN or CAR

PEs bearing various antibacterial groups can be used to form various functional materials with prolonged antimicrobial activity over time and sufficient biodegradability. In the wide range of antimicrobial agents, the substances of plant origin show highly effective antibacterial activity.^10^ Among them, essential oils including THY, MEN and CAR are promising bioactive compounds with reduced systemic toxicity. The synthesis of new PEs-DAF containing naturally derived antibacterial agents chemically coupled to the PE backbone through the labile bonds is a novel strategy for obtaining antimicrobial polymeric materials.

PAA decorated with THY, MEN or CAR was synthesized under mild conditions using Steglich esterification, DCC as coupling agent, and DMAP as catalyst (Scheme 1). PAA was functionalized with different degrees of substitution (DS) of essential oil groups, abbreviated as PAA-X-DS% (X = THY, MEN, CAR; DS = 5, 15) (Table 1).

**Scheme 1:**
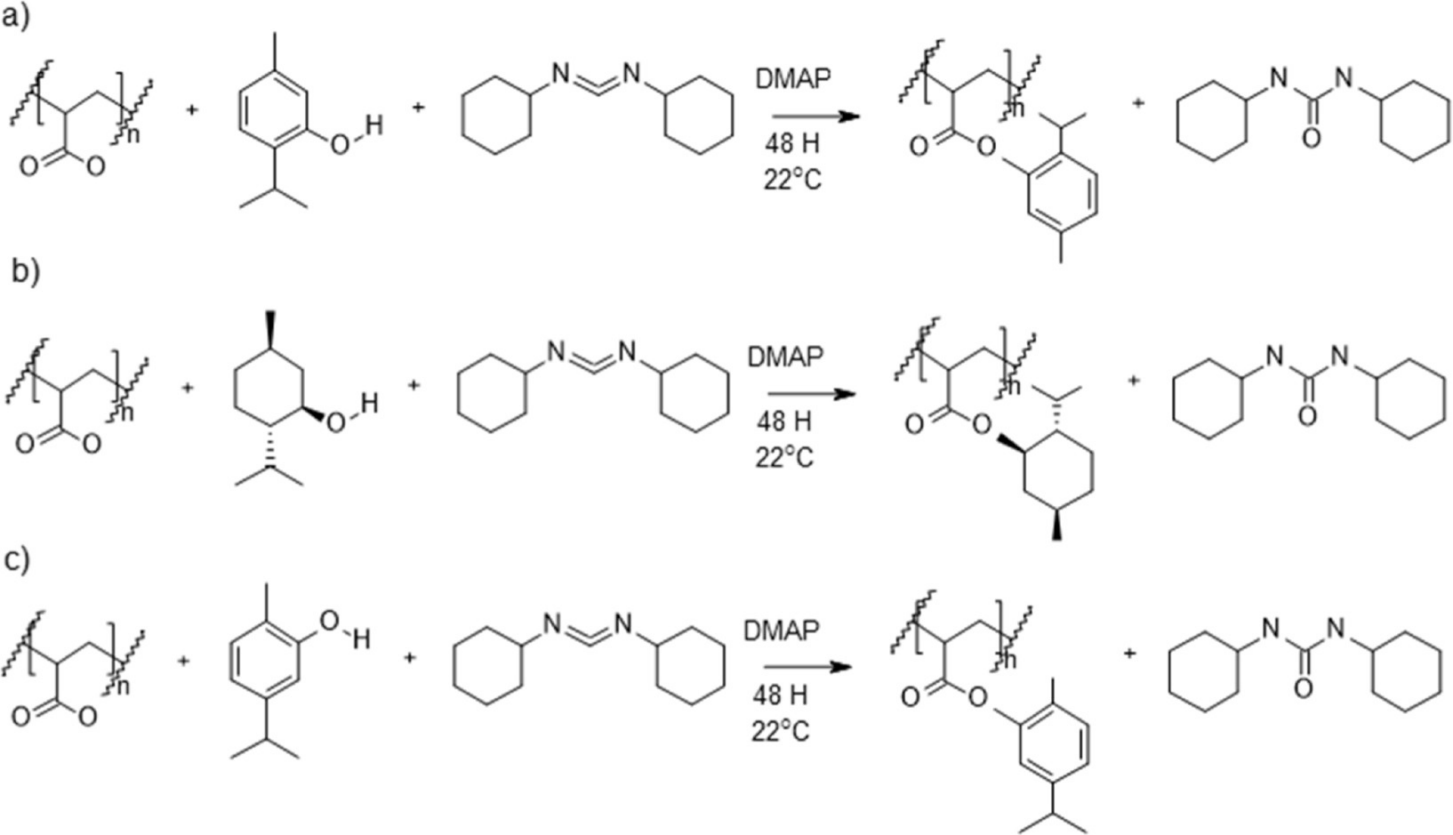
Synthesis of PAA Functionalized with (a) THY, (b) MEN, and (c) CAR Obtained by Steglich Esterification

**Table 1:**
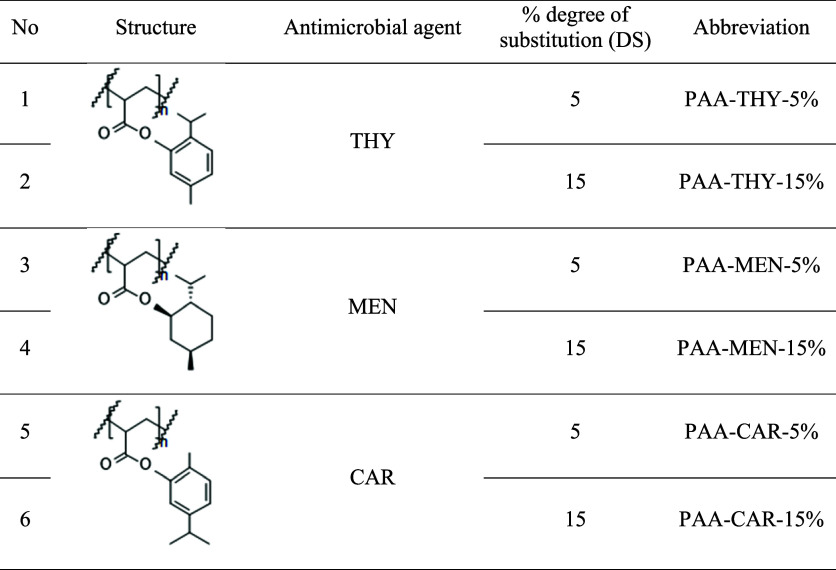
Structures and Abbreviations of the PAA Modified with THY, MEN, or CAR

The chemical structures of the obtained products were confirmed by 1H NMR analysis (see Figure 1). In all spectra, signals attributed to the polymer backbone (broad peaks between 1 ppm and approximately 2.5 ppm)^11^ and essential oil side motifs (sharp peaks at various locations between 0.8 ppm and approximately 7 ppm) are evident.^12^ Notably, unlike THY and CAR, MEN contains solely aliphatic/alicyclic groups. Therefore, the spectra of PAA-MEN-5% and PAA-MEN-15% do not display any signals at chemical shifts exceeding approximately 4 ppm. Moreover, the 1H NMR spectra are in accordance with the degrees of substitution for the specific products. The relative integrated intensities between the aromatic signals (strong, sharp multiplets at 6.5–7 ppm) and the aliphatic polymer backbone (three broad signals at 1.2–2 ppm and around 2.5 ppm) are three times higher for PAA-CAR-15% compared to PAA-CAR-5%. Similarly, for PAA-THY-15%, the relative integrated intensities between the aromatic signals (three sharp multiplets at 6.5–7 ppm) and the aliphatic polymer backbone (two broad signals at 1.75 and 2.25 ppm) are three times higher compared to PAA-THY-5%. As MEN contains no aromatic motifs, the degree of substitution can be estimated by comparing isolated aliphatic signals at 0.8 ppm (two methyl groups in the MEN motif) with three broad signals at chemical shifts of 1.2–2.5 ppm (polymer backbone). Thus, the relative intensities (aliphatic signals at 0.8 ppm to broad peaks at 1.2–2.5 ppm) for PAA-MEN-15% are three times higher than for PAA-MEN-5%.

**Figure 1:**
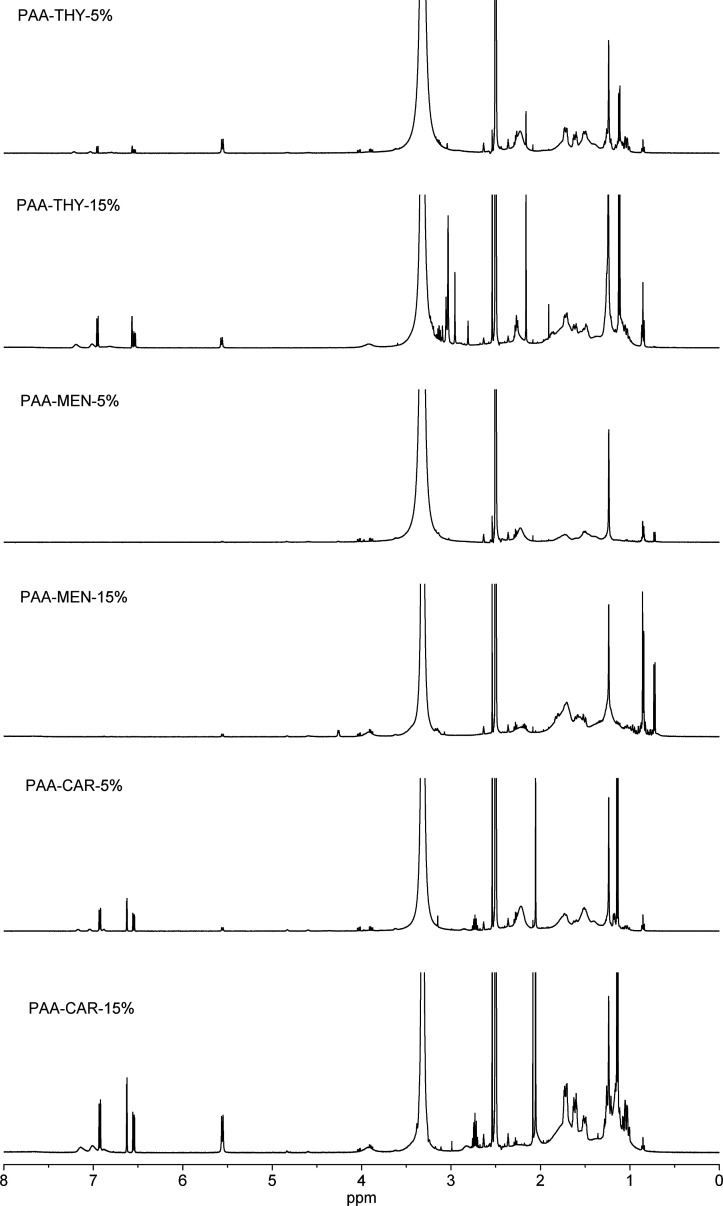
^1^H NMR spectra of the obtained products (PAA-THY-5%, PAA-THY-15%, PAA-MEN-5%, PAA-MEN-15%, PAA-CAR-5% and PAA-CAR-15%) dissolved in DMSO-*d*_6_.

The outcomes of the ^1^H NMR analysis demonstrate a high degree of correlation between the proposed structures and the synthesized products. Additionally, notable distinctions are evident between the products, which vary in degree of substitution (5% or 15%), confirming the versatility of the employed synthetic methodologies.

The chemical structures of the functionalized PEs were also confirmed by Fourier transform infrared (FTIR) spectroscopy. The spectra of this analysis and their description are presented in the electronic Supporting Information (ESI) in Figure S1.

### 2.2 Characterization of Hydrogel Microparticles Decorated by Functional Coatings

The hydrogel microparticles were prepared in two stages. Initially, an emulsion method was employed to fabricate ALG-based microspheres, and the emulsion was cross-linked with calcium ions. Subsequently, the LbL technique was utilized to create microparticles coated with CHIT, which constituted the first layer and PAA decorated by antimicrobial essential oil (for further details, please refer to Table 1: PAA-THY-DS; PAA-MEN-DS; PAA-CAR-DS; DS = 5, 15%), as the outer functional layer, as illustrated in Figure 2. The objective was to produce microparticles comprising diverse outer PE coatings with antimicrobial properties. For that purpose, six types of microparticles were fabricated, differing in their PE shells and compositions. The details of these microparticles, including their abbreviations and characterization, are presented in Table 2.

**Figure 2:**
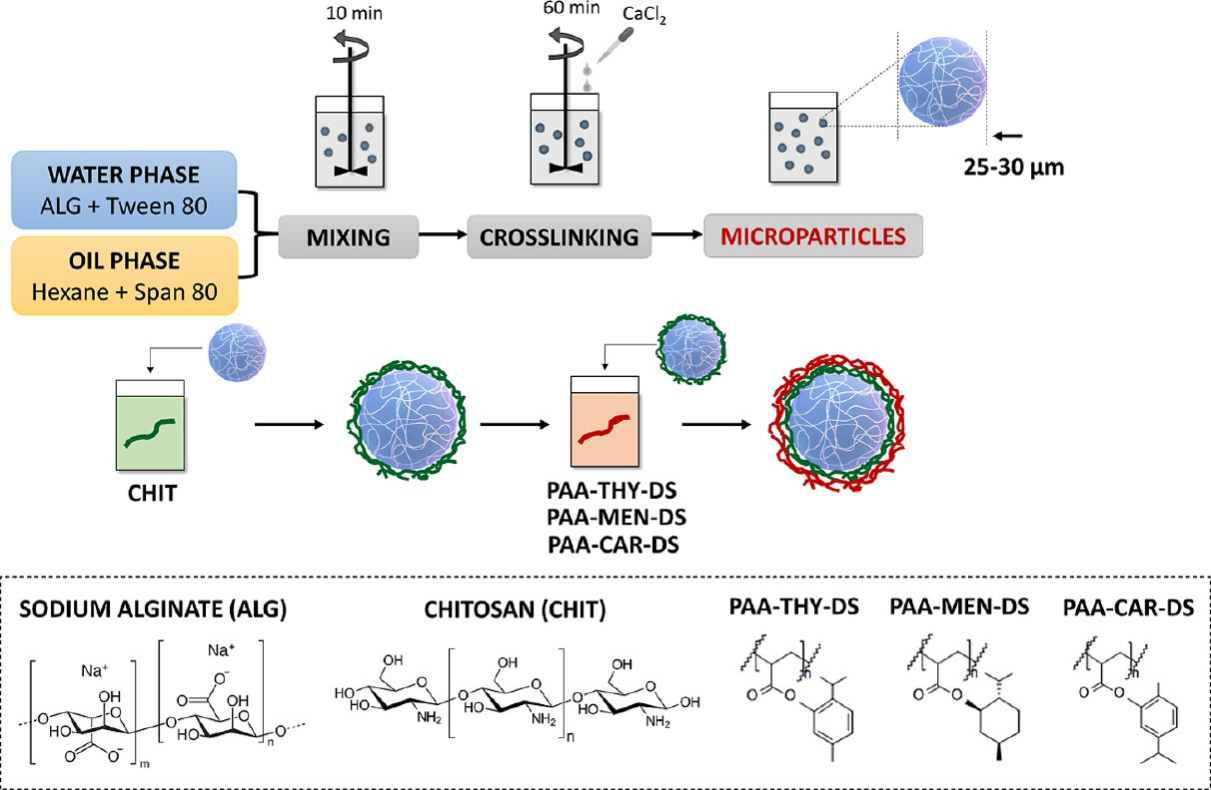
Scheme of the fabrication of hydrogel microparticles functionalized with PAA decorated by THY, MEN or CAR (denoted as PAA-THY-DS; PAA-MEN-DS; PAA-CAR-DS; DS = 5, 15%).

**Table 2:** Characterisation of Hydrogel Microparticles Functionalized with Antimicrobial Coatings^a^

core	coating	abbreviation	MD [μm]	PDI
ALG	CHIT/PAA-THY-5%	MT5	25.8 ± 3.8	0.021
	CHIT/PAA-THY-15%	MT15	29.1 ± 4.9	0.028
	CHIT/PAA-MEN-5%	MM5	27.9 ± 4.6	0.027
	CHIT/PAA-MEN-15%	MM15	28.5 ± 4.9	0.030
	CHIT/PAA-CAR-5%	MC5	26.1 ± 2.9	0.012
	CHIT/PAA-CAR-15%	MC15	27.1 ± 4.2	0.024

a MD—mean diameter. PDI—polydispersity index.

The mean diameter (MD) of the functionalized microparticles obtained was in the range of 25–30 μm, as illustrated in Table 2. In general, the type of outer functional coating did not affect the particle size, as no significant difference was observed among all the microparticles under study. The polydispersity index (PdI) values of the microparticles were below 0.3, indicating that their population could be considered monodisperse. Scanning electron microscopy (SEM) was employed to examine the shape and surface morphology of the dried microparticles, with representative micrographs presented in Figure 3. The investigation demonstrated that all functionalized microparticles exhibited a spherical shape. It was evident from the SEM images that the fabricated particles had a highly porous, wrinkled, and irregular surface, irrespective of the type of outer PE layer. These findings indicated that the kind of functional coating had no significant impact on the size and morphology of the microparticles. The microparticles observed in SEM had a size of approximately 20–30 μm, which was in alignment with the MD values obtained using the polarization microscope.

**Figure 3:**
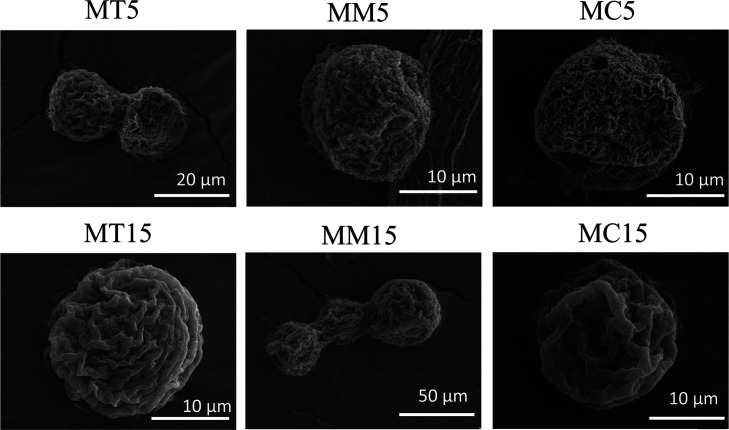
SEM images of hydrogel microparticles functionalized with antimicrobial coatings.

The decorated microparticles were studied by FTIR, and the most characteristic peaks reflecting structural properties and confirming the successful formation of the functionalized PAA were shown and described in Figure S2 in the ESI.

### 2.3 Characterization of Hydrogel Nanoparticles Decorated by Functional Coatings

The fabrication of hydrogel nanoparticles was also conducted in two stages. Initially, the high-pressure homogenization method was employed to create ALG-based nanospheres, which were cross-linked by adding calcium ions. Subsequently, the LbL technique was utilized to construct nanoparticles, which were coated with CHIT, as the initial layer and antimicrobial decorated PAA (for further details, please refer to Table 1: PAA-THY-DS; PAA-MEN-DS; PAA-CAR-DS; DS = 5, 15%), as the external functional layer, as illustrated in Figure 4. Six distinct types of nanoparticles were produced, each with a different antimicrobial PE shell. The variations in composition, abbreviations, and characterization are presented in Table 3. The mean diameter (MD) of the functionalized nanoparticles was observed to range from 123 to 165 nm, as illustrated in Table 3. The NT5 nanoparticles exhibited the lowest mean diameter (123 nm), while the NC15 nanoparticles demonstrated the greatest diameter (165 nm). The effect of the density of substitution was noticeable only for THY functionalized nanoparticles as their size increased from 123 to 165 nm. The size of MEN and CAR functionalized nanoparticles was the largest, practically independent of the DS.

**Figure 4:**
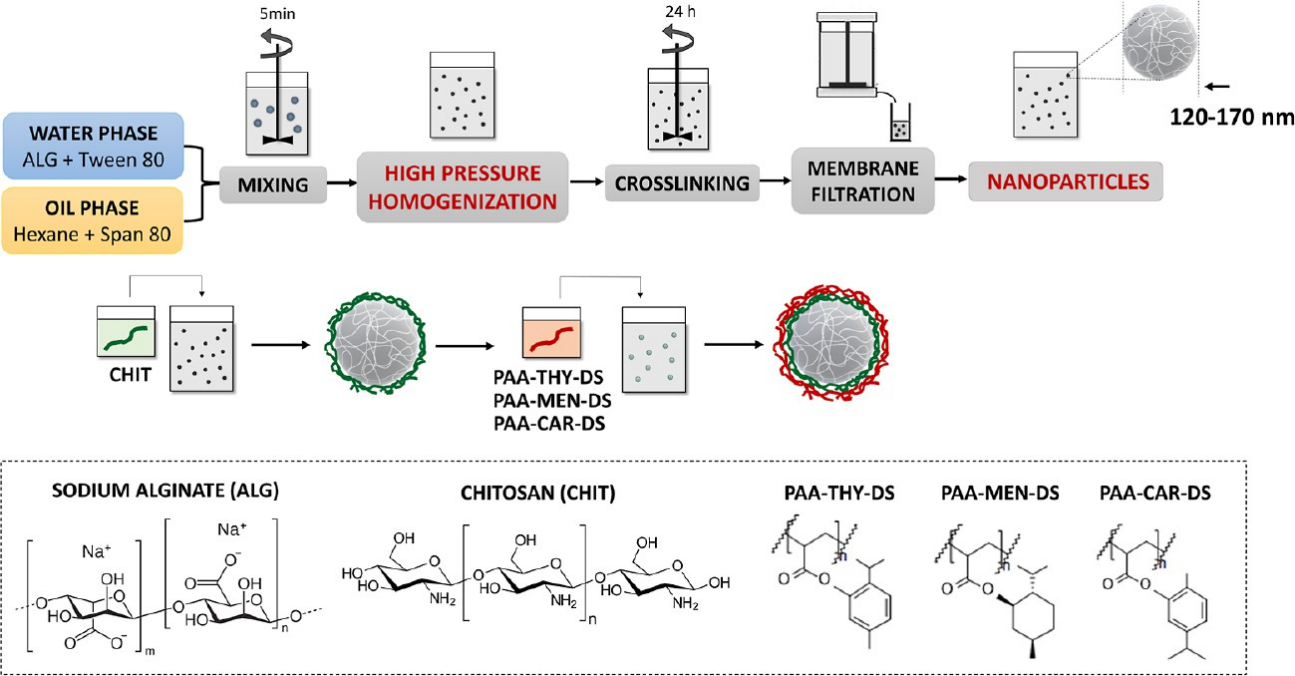
Scheme of the fabrication of hydrogel nanoparticles functionalized with PAA decorated by THY, MEN or CAR (denoted as PAA-THY-DS; PAA-MEN-DS; PAA-CAR-DS; DS = 5, 15%).

**Table 3:** Characterisation of Hydrogel Nanoparticles Functionalized with Antimicrobial Coatings^a^

core	coating	abbreviation	MD [nm]	PDI
ALG	CHIT/PAA-THY-5%	NT5	123 ± 8	0.276 ± 0.019
	CHIT/PAA-THY-15%	NT15	165 ± 9	0.310 ± 0.016
	CHIT/PAA-MEN −5%	NM5	152 ± 7	0.098 ± 0.016
	CHIT/PAA-MEN −15%	NM15	148 ± 3	0.304 ± 0.043
	CHIT/PAA-CAR −5%	NC5	158 ± 3	0.053 ± 0.029
	CHIT/PAA-CAR −15%	NC15	165 ± 6	0.336 ± 0.039

a MD—mean diameter. PDI—polydispersity index.

The polydispersity index (PdI) values of the NT5, NM5, and NC5 systems were below 0.3, thereby confirming the monodispersity of these nanoparticles. The polydispersity of the nanoparticles’ size was slightly increased for the higher degree of substitution (NT15, NM15, and NC15 systems), exceeding 0.3. The shape and surface morphology of the nanoparticles were investigated using transmission electron microscopy (TEM), and images of the studied nanoproducts are presented in Figure 5. As observed in the TEM photographs, the nanoparticles exhibited a regular spherical shape and a porous, rugged surface, irrespective of the type of functional shell. The nanoparticles had a size of approximately 100–200 nm, which agrees with the values obtained by DLS analysis. AFM images (see Figure 6) revealed rough spherical objects of ca. 200–300 nm, indicating the shape of the studied nanoparticles (NT15, NM15, and NC15—see Table 3). Larger objects may be attributed to aggregates of nanoparticles, possibly formed during samples’ drying.

**Figure 5:**
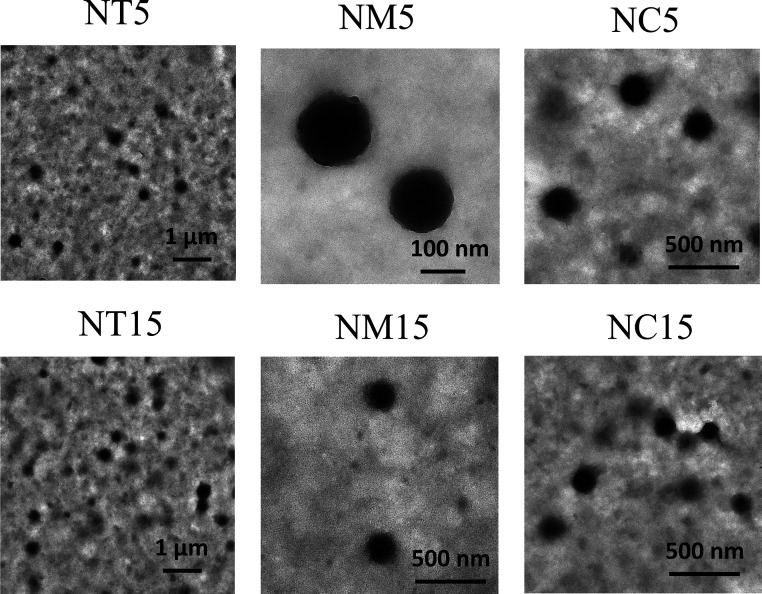
Transmission electron microscopy images of hydrogel nanoparticles functionalized with antimicrobial coatings.

**Figure 6:**
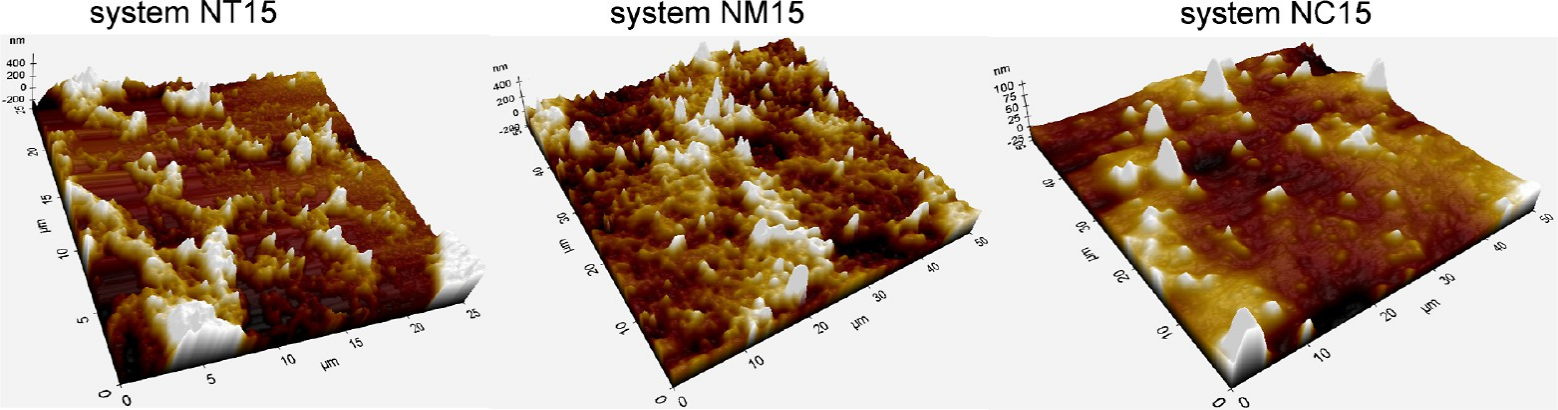
AFM images of hydrogel nanoparticles functionalized with antimicrobial coatings.

The decorated nanoparticles were studied by FTIR, and their spectra were presented and described in Figure S3 in the ESI.

The PE build-up onto the nanoparticles was confirmed by alterations in their surface charge after each PE adsorption, as illustrated in Figure 7. The deposition of the initial CHIT layer resulted in a shift from a negative zeta potential (ζ = −38 mV) for uncoated alginate cores to a positive value (ζ = +54 mV). Figure 7 illustrates the typical zigzag dependence of the nanoparticles’ zeta potential following the formation of subsequent PE shells, indicating the assembly of new functionalized PEs on the nanoparticle cores. Following the adsorption of the second functional coating, the surface charge was observed to reverse, with values of zeta potential −23 mV for PAA-THY-5%, −36 mV for PAA-THY-15%, and −35 mV for PAA-MEN-5%, −37 mV for PAA-MEN-15%, −22 mV for PAA-CAR-5%, and −39 mV for PAA-CAR-5%—coated nanoparticles. Furthermore, the surface charge of the NT15, NM5, NM15, and NC15 nanoparticles was sufficiently high for electrostatic stabilization. Moreover, according to AFM imaging, sample NC15 exhibited the lowest tendency to aggregate at the surface, most likely due to the most negative value of zeta potential among the studied samples. The observed increase in negative zeta potential for a higher degree of substitution of PAA with THY and CAR, i.e., for nanoparticles coated with less negative polyanion, may be attributed to the difference in the conformation of the polymer coating with the increased grafting of those moieties.

**Figure 7:**
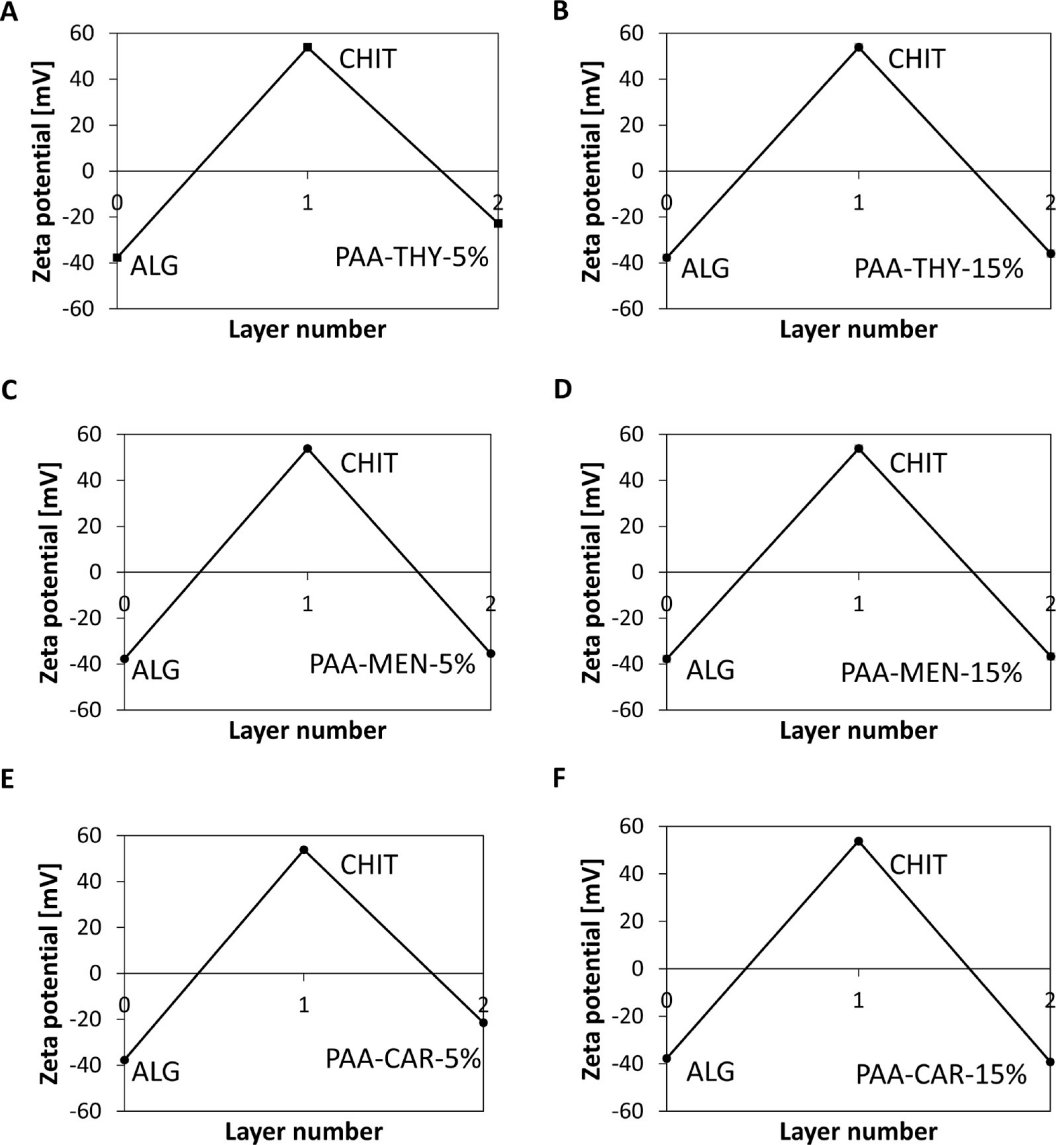
Zeta potential changes upon adsorption of functional PE layers: CHIT/PAA-THY-5% (A), CHIT/PAA-THY-15% (B), CHIT/PAA-MEN-5% (C), CHIT/PAA-MEN-15% (D), CHIT/PAA-CAR-5% (E), CHIT/PAA-CAR-15% (F) on the nanoparticle surface.

### 2.4 Physicochemical Properties of LbL Coatings

The QCM-D technique is particularly well suited for determining adsorption kinetics and investigating the mechanism of PE build-up as it facilitates the real-time monitoring of the PE layers formation process.^13^ Accordingly, this method was employed to gain a comprehensive insight into the deposition of novel functional polymeric coatings. In order to emulate the construction of the LbL coatings comprising nano- and microparticles, a series of functional PE films, including PAA-THY-5%, PAA-THY-15%, PAA-MEN-5%, PAA-MEN-15%, PAA-CAR-5%, and PAA-CAR-15%, were assembled on the surface of previously adsorbed PEI/ALG/CHIT layers. The findings regarding the deposition of PEI/ALG/CHIT coatings were presented in our previous paper.^15^ Figure 8 illustrates the profiles of the frequency (Δ*f*) and dissipation energy (Δ*D*) shifts variations upon PE adsorption. The typical decrease in frequency shift indicates that mass was added to the surface, while the increase in dissipation signal denotes the formation of soft and viscoelastic films. Conversely, upon rinsing the QCM sensor with distilled water, a slight increase in frequency and a decrease in dissipation to a lower value are observed, which can be attributed to the removal of excess PE. The dissipation values exceeding 1 × 10^–6^ per 10 Hz frequency variation and separated overtones indicate the formation of soft and flexible coatings.^13^

**Figure 8:**
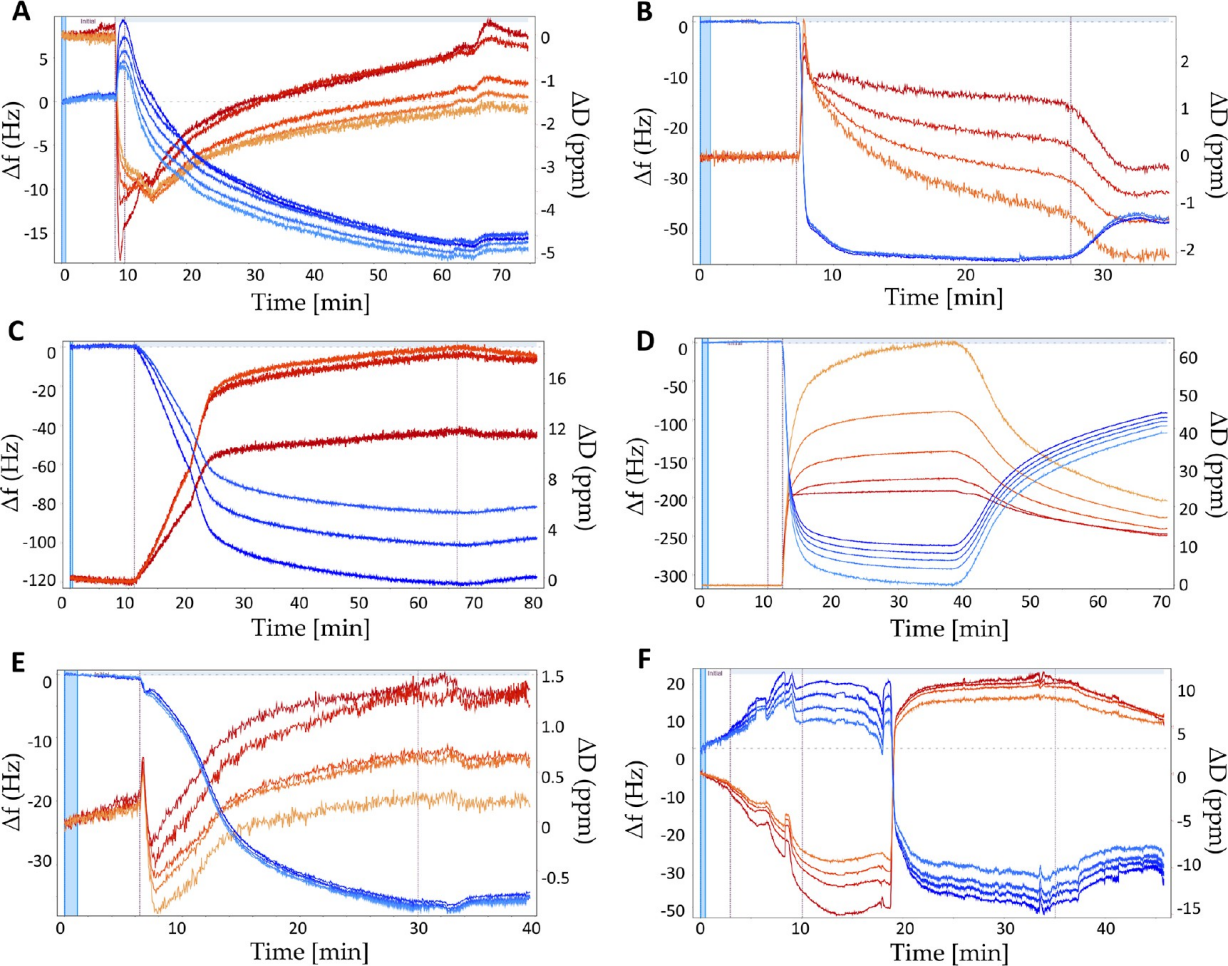
QCM-D graphs presenting the frequency and dissipation shifts as a function of time during the buildup of PE films: (A) PAA-THY-5%, (B) PAA-THY-15%, (C) PAA-MEN-5%, (D) PAA-MEN-15%, (E) PAA-CAR-5%, (F) PAA-CAR-15% adsorbed on PEI/ALG/CHIT coatings.

The QCM-D results demonstrated the successful deposition of all the studied PEs as functional coatings on PEI/ALG/CHIT layers. The assembly of PEI/ALG/CHIT coating was described in detail in our previous works.^11,15^ In these articles, the comparison of PEI, ALG, and CHIT layers showed the strongest adsorption of CHIT films and the weakest deposition of PEI films. It is worth noting that the assembly of PAA-MEN-5% and PAA-MEN-15% resulted in a marked shift in frequency compared to the values obtained during the adsorption of the other functionalized PE shells. Similar frequency variations were observed during the deposition of coatings composed of PAA modified with THY (PAA-THY-5%, PAA-THY-15%) and CAR (PAA-CAR-5%, PAA-CAR-15%). Moreover, the profiles of frequency changes indicated that the assembly of PAA-THY-15%, PAA-MEN-15%, and PAA-CAR-15% induced a more significant frequency shift than PAA-THY-5%, PAA-MEN-5%, and PAA-CAR-5%, respectively. This outcome demonstrated that PAAs decorated with a higher degree of substitution of the antimicrobial agent adsorb in higher quantities on the PEI/ALG/CHIT layers than those with lower degrees of substitution.^14^

The viscoelastic properties of the PE coatings under investigation were also elucidated through QCM-D measurements. Referring to our previous paper,^15^ the results indicated that ALG formed soft layers, while PEI and CHIT gave rise to rather stiff films. Following the assembly of the PAA-THY-5%, PAA-THY-15%, PAA-CAR-5%, and PAA-CAR-15% layers, the dissipation energy values were observed to be below 1 × 10^–6^ or slightly above 1 × 10^–6^, suggesting the formation of films with a high degree of rigidity. These findings are consistent with those previously reported,^11^ where we demonstrated that the adsorption of PAA resulted in the stiffening of the film, leading to the formation of a rigid and thin structure. In contrast, the formation of PAA-MEN-5% and PAA-MEN-15% layers resulted in a notable increase in dissipation values and their spread for the oscillation overtones, leading to the formation of viscoelastic films.

The QCM-D analysis enabled the determination of the physicochemical parameters of functional PE coatings, including the thickness, areal mass, viscosity, and elastic modulus. Their values for the considered functional coatings are collected in Table 4. Comparing PEI/ALG/CHIT layers, CHIT formed the thickest film, while PEI made the thinnest one. Moreover, the viscoelasticity parameters proved that ALG led to the construction of soft layers, in contrast to PEI and CHIT, which formed more rigid ones. The analyses of functional coatings confirmed the formation of thicker and more viscoelastic films with MEN-modified PAA than THY-modified or CAR-modified PAA. Similarly, the mass of each MEN-modified PAA film deposited on the PEI/ALG/CHIT coating was greater than that of the THY-modified or CAR-modified PAA film. However, the comparison of the thickness of those films determined by QCM-D (wet conditions) and spectroscopic ellipsometry (dry film) indicates that PAA-THY-DS films are much more hydrated and swollen in the wet state. In other systems, the thickness of dry and wet films was similar. A similar conclusion can be drawn from the analysis of the elasticity parameter of the coatings. The deposition of a MEN-functionalized PAA film resulted in significantly lower elastic modulus values than those obtained after the deposition of THY-modified or CAR-modified PAA films, irrespective of the degree of substitution of the active substance. These findings suggest that the adsorption of MEN-decorated PAA layers contributes to the formation of soft coatings, whereas the deposition of THY- or CAR-decorated PAA layers results in the construction of rigid coatings. In the case of THY-modified PAA layers, spectroscopic ellipsometry demonstrated that PAA-THY-15% formed a thinner film than PAA-THY-5%, which was inverse to that observed in QCM-D measurements. This phenomenon may be attributed to the interpenetration of the dry PAA-THY-15% layer, which results in reduced residual hydration and, consequently, a reduction in thickness. That can partially explain the increase in the negative zeta potential with the density of substitution by THY moieties.

**Table 4:** Characteristics of the PE coatings determined using the QCM-D and spectroscopic ellipsometry analysis

PE coating	quartz crystal microbalance with an energy dissipation				spectroscopic ellipsometry
	thickness [nm]	areal mass [μg/cm2]	viscosity [mPa/s^–1^]	elastic modulus [kPa]	thickness [nm]
PEI	2.9 ± 0.7	0.3 ± 0.1	2.5 ± 0.3	160.9 ± 0.5	0.9 ± 0.1
ALG	7.8 ± 1.4	0.8 ± 0.1	3.2 ± 0.7	151.8 ± 0.2	3.7 ± 0.4
CHIT	28.5 ± 2.0	2.9 ± 0.3	2.4 ± 0.4	264.5 ± 0.4	2.1 ± 0.2
PAA-THY-5%	2.5 ± 1.2	0.3 ± 0.1	3.8 ± 0.6	184.6 ± 0.8	6.7 ± 0.1
PAA-THY-15%	5.8 ± 1.7	0.6 ± 0.2	2.6 ± 0.2	202.6 ± 0.5	3.5 ± 0.1
PAA-MEN −5%	20.0 ± 1.3	2.0 ± 0.2	1.9 ± 0.3	2.6 ± 0.3	6.0 ± 0.1
PAA-MEN-15%	47.9 ± 2.4	5.0 ± 0.4	1.7 ± 0.2	1.1 ± 0.1	7.2 ± 0.1
PAA-CAR-5%	6.8 ± 0.9	0.7 ± 0.1	2.7 ± 0.5	131.2 ± 0.4	7.5 ± 0.3
PAA-CAR-15%	7.3 ± 1.1	0.7 ± 0.1	1.2 ± 0.1	54.8 ± 0.2	12.8 ± 0.1

MEN-functionalized PAA constructs generally exhibited softer coatings than those functionalized with THY or CAR. This phenomenon can be attributed to the differing structural characteristics of the bioactive agents under investigation. The cyclohexyl ring in MEN provides the structure with molecular and spatial elasticity, thereby facilitating dynamic and flexible interactions with the PE chains. However, the more rigid aromatic rings present in THY and CAR restrict their ability to form flexible interactions with PAA, resulting in forming PE coatings with higher stiffness.^16^ Additionally, π–π stacking of aromatic rings can contribute to more compact and stiff structures.

### 2.5 Antimicrobial Studies

The decoration of hydrogel nano- and microparticles with polyelectrolyte complexes containing essential oil-based moieties has been demonstrated to confer antimicrobial properties, thereby protecting against bacterial proliferation. These structures can potentially serve as multifunctional drug delivery systems (DDSs), thereby enhancing the intended biological activity. The antimicrobial characteristics of these structures are susceptible to several factors. (i) The nature of functionalized PEs; (ii) the chemical composition of particle coatings; (iii) the type of bacterial cell wall; (iv) the environmental conditions. The present study was conducted to investigate the antibacterial activity of functionalized PAAs, nano- and microparticles decorated with these PEs, as well as THY, MEN and CAR, which were employed as standard antimicrobial agents against S. aureus and E. coli. That was achieved through the utilization of agar disc-diffusion assays and minimal inhibitory concentration (MIC) evaluations. The results of the disc-diffusion method, expressed as inhibition zone diameters (Φ), are presented in Table 5 and illustrated in Figure 9. The results of the MIC evaluations are presented in Table 6.

**Table 5:** Diameters of Inhibition Zone (mm) for Functionalized PEs, Nanoparticles and Microparticles Decorated by These PEs Against S. aureus and E. coli^a^

	studied sample	zone of inhibition diameter (mm)	
		S. aureus	E. coli
antimicrobial agent	THY	15.5 ± 1.1	14.8 ± 0.3
	MEN	14.3 ± 0.9	14.4 ± 0.6
	CAR	12.3 ± 0.5	12.5 ± 1.5
polyelectrolyte	control	10.5 ± 0.5	8.0 ± 0.1
	PAA	9.0 ± 0.8	9.0 ± 0.8
	PAA-THY-5%	12.7 ± 1.4	10.2 ± 1.1
	PAA-THY-15%	15.5 ± 0.6	15.3 ± 0.4
	PAA-MEN −5%	14.1 ± 1.0	10.1 ± 1.3
	PAA-MEN-15%	12.5 ± 0.7	11.4 ± 0.9
	PAA-CAR-5%	11.4 ± 0.5	11.0 ± 1.2
	PAA-CAR-15%	13.6 ± 0.4	12.3 ± 1.5
microparticle	control	9.0 ± 0.2	8.0 ± 0.3
	MCHIT	10.6 ± 0.5	10.7 ± 0.6
	MT5	17.4 ± 0.9	14.1 ± 1.0
	MT15	18.2 ± 1.4	13.4 ± 0.8
	MM5	16.5 ± 0.8	11.1 ± 0.4
	MM15	17.1 ± 1.2	14.3 ± 0.5
	MC5	14.0 ± 0.6	10.8 ± 0.3
	MC15	14.5 ± 0.3	11.7 ± 0.4
nanoparticle	control	8.0 ± 0.2	9.2 ± 0.3
	NCHIT	8.0 ± 0.1	9.5 ± 0.2
	NT5	8.5 ± 0.4	11.5 ± 0.5
	NT15	12.0 ± 0.3	16.5 ± 0.4
	NM5	8.0 ± 0.6	14.5 ± 1.0
	NM15	12.5 ± 1.1	15.0 ± 1.2
	NC5	11.0 ± 0.5	11.5 ± 0.6
	NC15	12.5 ± 0.9	11.5 ± 0.5

a MCHIT—microparticles coated with CHIT. NCHIT—nanoparticles coated with CHIT.

**Figure 9:**
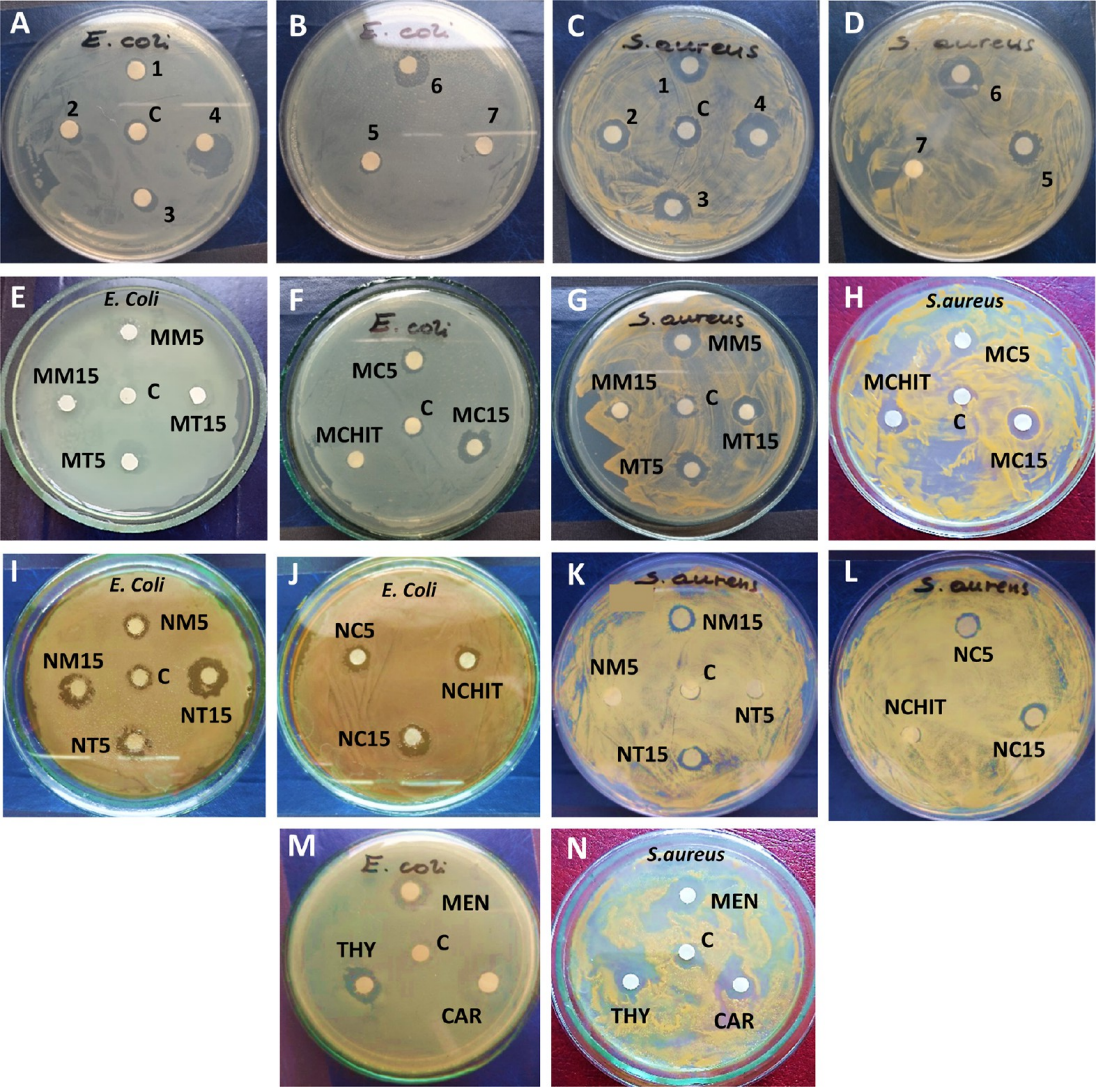
Photographs of sample inhibition zone test plates for decorated PEs: PAA-MEN-5% (1), PAA-MEN-15% (2), PAA-THY-5% (3), PAA-THY-15% (4), PAA-CAR-5% (5) PAA-CAR-15% (6) and PAA (7) (A–D), as well as microparticles MCHIT, MT5, MT15, MM5, MM15, MC5, MC15 (E–H) nanoparticles NCHIT, NT5, NT15, MM5, NM15, NC5, NC15 (I–L) and bioactive agents such as THY, MEN, CAR (M,N) against S. aureus and E. coli. Ethanol was used as a control, C.

**Table 6:** Minimal Inhibitory Concentrations (μg/mL) for Functionalized PEs and Nanoparticles Decorated by These PEs Against S. aureus and E. coli^a^

	studied sample	minimal inhibitory concentrations (μg/mL)	
		S. aureus	E. coli
antimicrobial agent	THY	400.0	400.0
	MEN	3200.0	800.0
	CAR	400.0	200.0
polyelectrolyte	PAA-THY-15%	400.0	312.5
	PAA-MEN-15%	>5000.0	625.0
	PAA-CAR-15%	625.0	625.0
nanoparticle	NCHIT	>5000	>5000
	NT15	2500	312.5
	NM15	>5000	625.0
	NC15	625.0	625.0

a MCHIT—microparticles coated with CHIT. NCHIT—nanoparticles coated with CHIT.

The disc-diffusion assay demonstrated that standard agents, including THY, MEN and CAR, exhibited notable antibacterial properties against both Gram-negative and Gram-positive bacteria. A comparison of the antimicrobial efficacy of the essential oils revealed that irrespective of the bacterial strain, THY demonstrated the highest efficacy, while CAR showed the lowest. Notably, the control sample and unmodified PAA exhibited minimal inhibitory effects, whereas PEs-DAF demonstrated a pronounced inhibitory impact on bacterial growth. As expected, the degree of substitution in the essential oil group affected the functionalized PEs’ antimicrobial activity. The samples PAA-THY-15%, PAA-MEN-15%, and PAA-CAR-15% exhibited larger zones of inhibition than the samples PAA-THY-5%, PAA-MEN-5%, and PAA-CAR-5%, respectively. Moreover, all modified PEs exhibited augmented antibacterial properties against S. aureus compared to E. coli. These findings agree with previously reported ones, wherein we similarly observed a more pronounced inhibitory effect against Gram-positive than Gram-negative bacteria.^11^ That allows for the hypothesis that the novel PEs-DAF representatives exhibit greater sensitivity toward Gram-positive bacteria. This phenomenon can be attributed to the distinctive structure of the bacterial cell, particularly the presence of an additional outer membrane in Gram-negative bacteria, which serves as a permeability barrier.^17^

The bioactivity analysis of microparticles decorated with novel PEs-DAF revealed an intriguing phenomenon. The microparticles coated with functionalized PEs demonstrated enhanced efficacy compared to native PEs in solution. This behavior can be attributed to the effect of PEs-DAF adsorption on the microparticles’ surface and the change of PEs conformation into an extended coil, which renders the antimicrobial essential oils moieties more accessible to bacteria. A similar phenomenon was previously described in the published work.^11^ Moreover, all the examined microparticles demonstrated superior bacterial inhibition against S. aureus compared to E. coli, analogous to the novel PEs-DAF. Conversely, the investigation of nanoparticles functionalized with PEs-DAF revealed an opposing effect. Here, the nanoparticles under examination demonstrated a greater capacity to inhibit bacterial growth in the case of E. coli than in the case of S. aureus. Furthermore, the nanoparticles NT15, NM5 and NM15 exhibited superior antibacterial properties against E. coli compared to the native PEs, including PAA-THY-15%, PAA-MEN-5% and PAA-MEN-15%.

The results of the disc-diffusion assay indicate that functionalized microparticles exhibit superior antimicrobial potential compared to nanoparticles. This phenomenon can be attributed to the small size of nanoparticles, which results in weaker adhesion to bacterial membranes, limiting the spatial proximity of individual antimicrobial groups and a subsequent reduction in their antibacterial efficacy.

Notably, the antimicrobial action of nano- and microparticles decorated with PAAs grafted with 15% of essential oil fragments was significantly enhanced compared to those with 5% substitution, irrespective of the bacterial strain.

The above analysis proved that all functionalized microparticles demonstrated higher antimicrobial activity against S. aureus than free essential oils. A similar effect was observed for certain nanoparticles, including NT15, NM5, and NM15, as they showed superior bacterial inhibition against E. coli compared to essential oils. The modification of particles’ surface with PEs-DAF allows for more efficient interaction with bacterial cells, thereby increasing membrane disruption and consequently enhancing their bactericidal effects. These results highlight the advantages of functionalized delivery platforms over conventional antimicrobial compounds in enhancing the antibacterial activity of essential oils and addressing challenges associated with their volatility and rapid degradation.

As the disk diffusion method is qualitative, an in-depth test was conducted to determine the minimum inhibitory concentration (MIC). The results of the disk diffusion assay indicated that the PAAs functionalized with 15% of essential oil moieties, as well as the nanoparticles decorated with such PEs, were the most promising systems and were therefore selected for further studies. The decorated microparticles were not subjected to the microdilution test, as it was not possible to obtain a homogeneous suspension of the systems in the medium. Nevertheless, the microdilution assay and the disc-diffusion assay results were consistent.

The antimicrobial effect of monoterpenes, including MEN, THY and CAR, is comparable. In both Gram-positive bacteria (S. aureus) and Gram-negative bacteria (E. coli), disruption of the cell membrane function was observed. The presence of a hydroxyl group increases the hydrophilicity of the compounds, thereby facilitating their dissolution and penetration of the cell membrane, which ultimately results in damage. Furthermore, the hydroxyl group acts as a protonophore, altering the physical and chemical properties of the membrane and contributing to the high antimicrobial activity of these compounds.^18–20^

In our studies, MEN and CAR demonstrated higher activity against E. coli with MICs of 0.8 and 0.2 mg/mL, respectively. That may be attributed to the strongly negative charge of the cell membrane of these bacteria, which is a consequence of the presence of a lipopolysaccharide layer. Furthermore, THY demonstrated activity against Gram-negative bacteria (MIC = 0.4 mg/mL). These findings corroborate those obtained in the disc-diffusion assay, as an increase in both the MIC differences and the inhibition zone diameters was observed concurrently. The reduced efficacy of MEN (MIC = 3.2 mg/mL) and CAR (MIC = 0.4 mg/mL) against S. aureus may be attributed to a thick peptidoglycan layer, which may present a greater challenge for these essential oils. This effect was not observed with THY (MIC = 0.4 mg/mL) despite Gram-positive bacteria’s simpler structure than Gram-negative bacteria.^21^ Compared to literature data, MICs of MEN against E. coli and S. aureus are often above the range tested^22,23^ or around 0.5 and 1 mg/mL, respectively.^24^ The results demonstrate that CAR exhibits higher activity toward the bacteria tested, with a MIC value range of 0.2 ^24^ to 7.6 mg/mL.^22^ In earlier works, THY demonstrated weaker (MIC = 7.5 mg/mL; MIC = 15.1 mg/mL)^22^ or comparable activity toward E. coli and S. aureus to that observed in our studies (MIC = 0.3 mg/mL; MIC = 0.3 mg/mL).^23^ The discrepancies between the results can be attributed primarily to the disparate strains employed in the study, as well as the slightly divergent methodology.

The bioactivity of functionalized PEs was analyzed, and it was observed that PAA-THY-15% exhibited superior antibacterial properties against both bacterial strains in comparison to PAA-MEN-15% and PAA-CAR-15%. A similar phenomenon was observed during the disc-diffusion assay. Moreover, nanoparticles functionalized with PEs-DAF, such as NT15, NM15 and NC15, demonstrated antimicrobial activity against both bacteria, in contrast to CHIT-coated nanoparticles, which exhibited MIC values above 5 mg/mL. It is noteworthy that NT15 exhibited the highest level of activity among the nanoparticles tested against E. coli (MIC = 0.31 mg/mL) and, along with NM15, demonstrated superior antibacterial properties against E. coli compared to S. aureus. It has been demonstrated that the hydroxyl groups of essential oils are the key structural elements involved in the antimicrobial activity of these compounds. Consequently, some studied PEs and decorated particles revealed lower bioactivity than essential oils. However, the analysis above has shown that functionalized PEs and nano- and microparticles decorated by these PEs exhibit an inhibitory effect on the growth of both Gram-positive and Gram-negative bacteria. Our findings highlight the potential of antimicrobial functionalization of DDSs in overcoming bacterial resistance and improving treatment.

### 2.6 Encapsulation and Release of RES

The constructed hydrogel nano- and microparticles functionalized with antimicrobial PE coatings demonstrate considerable potential as controlled DDSs. Accordingly, the designed systems were loaded with RES, a model chemotherapeutic substance, to assess their applicable properties, including the ability to encapsulate and release a biologically active compound. The fabricated nano- and microparticles functionalized with PEs-DAF coatings may extend the group of multipurpose carriers by encapsulating an active ingredient in the designed systems.

#### 2.6.1 Encapsulation and Release of RES from Microparticles

The RES-loaded hydrogel microcarriers were prepared using the same techniques as those used to prepare the microparticles. Three types of RES-loaded microparticles decorated with the selected antimicrobial PE coatings were prepared, and their characterization is presented in Table 7. The dimensions of the microcarriers loaded with the bioactive compound ranged from 42 to 52 μm. It was observed that the RES-loaded microparticles were larger than the empty microparticles. These differences can be attributed to the fact that RES, as a hydrophobic substance, is dispersed as a suspension in the ALG aqueous solution, resulting in the formation of a larger microparticle core. The PDI values of the functionalized microcarriers indicate that the population can be considered as monodisperse. The obtained encapsulation efficiency (EE) values ranging from 60 to 70% showed that RES was effectively encapsulated in the decorated microcarriers. Microcarriers decorated with a PAA-CAR-15% layer proved to be the most effective for RES encapsulation (EE = 70%), while the decoration of microcarriers with a PAA-MEN-15% layer resulted in the least efficient RES loading (EE = 60%). However, the EE of RES for all microcarriers functionalized with antimicrobial PE coatings was satisfactory.

**Table 7:** Characterisation of RES-Loaded Hydrogel Microcarriers Functionalized with Antimicrobial Coatings^a^

payload	core	coatings	MD [μm]	PDI	EE [ %]
RES	ALG	CHIT/PAA-THY-15%	52 ± 6.5	0.015	68 ± 3
		CHIT/PAA-MEN-15%	42 ± 7.5	0.033	60 ± 3
		CHIT/PAA-CAR-15%	46 ± 6	0.020	70 ± 4

a MD—mean diameter. PDI—polydispersity index. EE—encapsulation efficiency.

The release profiles of the RES from microcarriers that have been functionalized with antimicrobial coatings are presented in Figure 10. They indicate that they exhibited a sustained and prolonged release of the bioactive compound. No significant difference in the release rate between the various systems was observed. The calculated time of 50% release of the active substance (*t*_0.5_) indicated that microcarriers with CHIT/PAA-THY-15% shells demonstrated the slowest release of the payload (*t*_0.5_ = 88 min). That can be attributed to the tightest structure of the shell, as demonstrated previously by QCM-D and ellipsometry results. Compared to the functionalized microparticles, the uncoated ones were characterized by the initial burst release of RES (*t*_0.5_ < 10 min). The antimicrobial-decorated PEs reduced burst release during the first 10 min from ∼50% to ∼15% for CHIT/PAA-THY-15%-coated carriers, ∼20% for CHIT/PAA-MEN-15%-coated carriers, and ∼30% for CHIT/PAA-CAR-15%-coated carriers. These studies revealed that the RES release rate can be controlled by the new functional PEs, applied as outer layers of microparticles.

**Figure 10:**
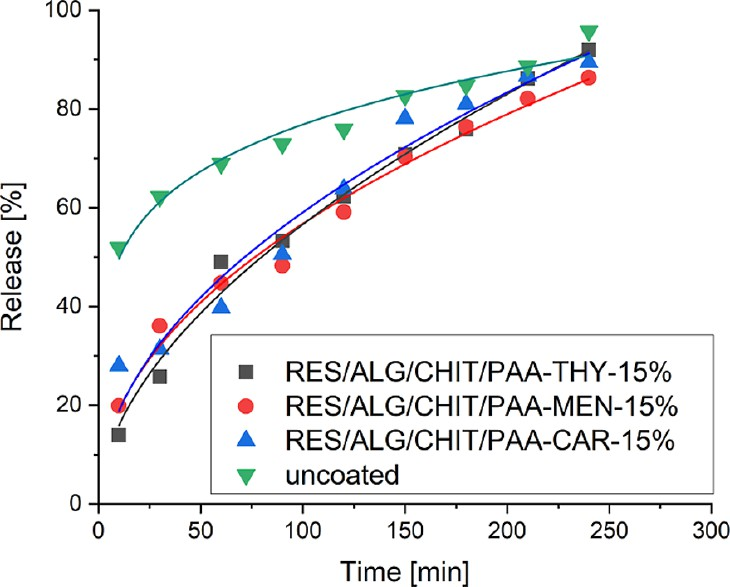
Release profiles of RES from microcarriers decorated by antimicrobial coatings in PBS at 37 °C.

The Korsmeyer–Peppas (KP) model was fitted to the experimental release data, and the resulting kinetic parameters are presented in Table 8. The KP model was successfully employed in our previous research,^25,26^ describing the release mechanism of natural substances from hydrogel microparticles. The calculated correlation coefficient (*R* ^2^) values exceeded 0.952 for all compositions of carrier systems, indicating a good fit of the selected model to the experimental data. The release mechanism of RES from the studied microparticles can be determined using the values of the release exponent coefficient (*n*): if *n* is less than 0.43, the payload release is characterized by Fickian diffusion; if 0.43 < *n* < 0.85, the release depends on non-Fickian or anomalous diffusion; if *n* > 0.85, the release is driven by the supercase II transport.^27,28^ The value of n for uncoated particles denotes that RES was released primarily through Fickian diffusion, indicating that the drug diffuses through the polymer matrix in a concentration-dependent manner. The obtained n values for all functionalized microsystems indicate that non-Fickian diffusion was the primary mechanism responsible for the release of RES. That indicates that the release of the active substance is governed by the diffusion and relaxation of the polymer chains in the particle core and polyelectrolyte shell, however, the diffusion dominates. The highest value of *n* for the CHIT/PAA-THY-15% layers indicates that the THY-decorated PAA adheres to the outer coatings of microparticles. In contrast, the lowest n value for CHIT/PAA-MEN-15% shells suggests that MEN-functionalized PAA forms more porous layers with favorable diffusion and release of RES that correlates with the QCM-D results.

**Table 8:** Kinetic Parameters Determined by Fitting the Korsmeyer–Peppas Model to the Data Pertaining to the Release of RES from Functionalized Microcarriers

	system			Korsmeyer–Peppas parameters		
payload	core	coatings	*t*_0.5_ [min]	*k*_m_ [min^–*n*^]	*n*	adj. *R* ^2^
RES	ALG	uncoated	<10	32.05 ± 2.34	0.19 ± 0.02	0.960
		CHIT/PAA-THY-15%	88	4.46 ± 0.69	0.55 ± 0.03	0.987
		CHIT/PAA-MEN-15%	81	6.40 ± 0.97	0.48 ± 0.03	0.982
		CHIT/PAA-CAR-15%	72	5.98 ± 1.52	0.50 ± 0.05	0.952

#### 2.6.2 Encapsulation and Release of RES from Nanoparticles

The hydrogel nanoparticles were loaded with RES using the HPH process and then decorated with antimicrobial PE shells using the LbL technique to form multipurpose nanocarrier systems. The results of the characterization of three types of RES-loaded nanocarriers functionalized with the selected antimicrobial PE shells are presented in Table 9. The mean diameter (MD) of the RES-loaded hydrogel nanoparticles ranged from 181 to 193 nm, slightly larger than that of the unloaded ones. The same was observed for the hydrogel microparticles coated with functional layers. The PdI values of RES-loaded nanocarriers appear to be below 0.3, indicating a monodisperse population. The calculated EE values were above 70%, confirming the efficient encapsulation of RES in the decorated nanoparticles. The loading of the drug in the functionalized hydrogel nanosystems was slightly more effective than in the microcarriers. The encapsulation of RES in the PAA-CAR-15% decorated nanoparticles seemed the most efficient (EE = 75%).

**Table 9:** Characterisation of RES-Loaded Hydrogel Nanocarriers Functionalized with Antimicrobial Coatings^a^

payload	core	coatings	MD [nm]	PDI	EE [ %]
RES	ALG	CHIT/PAA-THY-15%	185 ± 4	0.292 ± 0.008	70 ± 2.2
		CHIT/PAA-MEN-15%	182 ± 4	0.093 ± 0.016	72 ± 1.5
		CHIT/PAA-CAR-15%	193 ± 5	0.154 ± 0.026	75 ± 2.0

a MD—mean diameter. PDI—polydispersity index.

The release profiles of RES from the functionalized nanoparticles are presented in Figure 11. The kinetic curves of the active compound demonstrated similar release profiles. The calculated parameter *t*_0.5_ indicated that the highest release rate of RES was observed for PAA-MEN-15%-decorated nanocarriers (*t*_0.5_ = 53 min), while the slowest release rate of the payload was noticed for PAA-CAR-15%-modified nanoparticles (*t*_0.5_ = 69 min). Compared to the uncoated nanocarriers that demonstrated a fast release rate, the functionalized nanoparticles slowed down the release of the active compound, resulting in more sustained and prolonged release. In general, nanocarriers coated with antimicrobial layers exhibited faster initial release of RES, with a more pronounced burst effect, than surface-functionalized microparticles, followed by a slower release at longer times.

**Figure 11:**
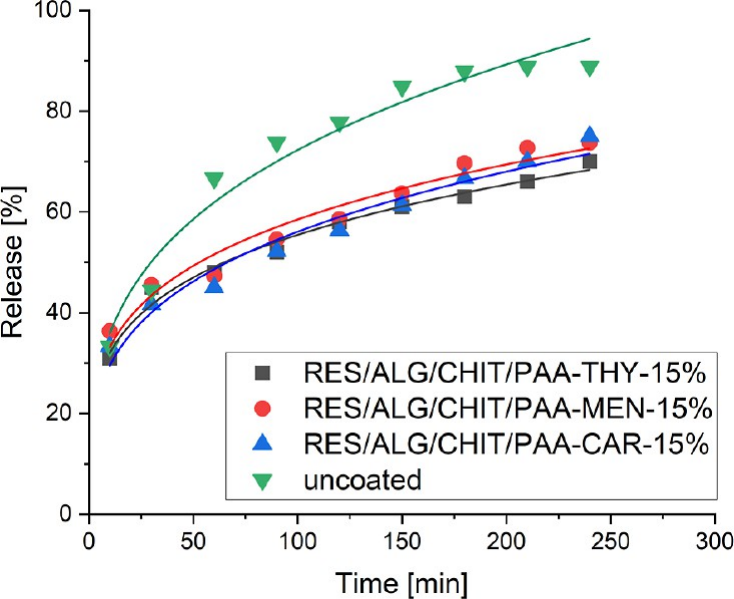
Release profiles of RES from nanocarriers decorated by antimicrobial coatings in PBS at 37 °C.

The Korsmeyer–Peppas model was selected to describe the release of RES from the nanocarriers. The selection of the kinetic model was based on the correlation coefficient (*R* ^2^) values. The *R* ^2^ values obtained demonstrated a good fit for the payload release by the KP model, with values exceeding 0.952 for all types of nanoparticles when the burst period was disregarded (see Table 10). The release exponent coefficient (*n*) values indicated that the drug release was driven by Fickian diffusion, suggesting a correlation between RES release and the swelling behavior of the nanoparticles,^28^ which facilitated the diffusion of the active substance through the functionalized PE coatings.^29^

**Table 10:** Kinetic Parameters Determined by Fitting the Korsmeyer–Peppas Model to the Data Pertaining to the Release of RES from Functionalized Nanocarriers

	system			Korsmeyer–Peppas parameters		
payload	core	coatings	*t*_0.5_ [min]	*k*_m_ [min^–*n*^]	*n*	adj. *R* ^2^
RES	ALG	uncoated	37	17.80 ± 2.40	0.30 ± 0.03	0.958
		CHIT/PAA-THY-15%	66	18.45 ± 1.26	0.24 ± 0.01	0.979
		CHIT/PAA-MEN-15%	53	18.76 ± 2.02	0.25 ± 0.02	0.952
		CHIT/PAA-CAR-15%	69	15.53 ± 1.81	0.28 ± 0.02	0.957

## 3 Conclusions

Six novel polyelectrolyte derivatives with antimicrobial functionality (PEs-DAF) were synthesized and formulated. These included poly­(acrylic acid) (PAA) functionalized with thymol (THY), menthol (MEN), and carvacrol (CAR). The antimicrobial-decorated PAA derivatives were identified as suitable building blocks for the fabrication of hydrogel core–shell particles. We fabricated and characterized nano- and microparticles consisting of a sodium alginate (ALG) core with a bilayer shell comprising biocompatible/biodegradable chitosan (CHIT) as the first cationic layer coating and an outer layer of synthesized antimicrobial-functionalized PAA. The specific type and composition of the new PEs-DAF shells significantly impacted the physicochemical and biological properties of the designed systems. In particular, the thickness and viscoelasticity of the PE coatings exhibited variation depending on the chemical structure of the antimicrobial-decorated PAA derivatives. The nano- and microparticles coated with essential oil-grafted PAA exhibited satisfactory antimicrobial activity against both Gram-positive (S. aureus) and Gram-negative (E. coli) bacteria, with some examples showing bioactivity exceeding that of the functionalized PAAs. In addition, the developed systems were shown to effectively encapsulate and release an active compound in a controlled manner. The functionalized nano- and microcarriers reduced burst release compared to the uncoated particles, resulting in sustained and prolonged release profiles.

In conclusion, the surface functionalization of nano- and microparticles with PAA decorated by natural-based essential oil fragments allowed the formation of new carriers with antimicrobial functionality. The drug encapsulation and release properties, as well as the effective antibacterial activity of the designed systems, render them potential candidates for a wide range of tailored therapeutic applications, particularly in the treatment of pathogenic infections. However, despite these promising results, several challenges need to be addressed before these systems can be implemented in clinical applications. An in vivo efficacy of the developed nano- and microparticles, as well as a deeper understanding of their interactions with human cells, tissues, and the microbiome, require further investigation to ensure their safety and minimize potential cytotoxic effects. Nonetheless, this work has shown that the surface functionalization of nano- or microcarriers with antibacterial PEs presents a promising strategy for the development of novel antimicrobial systems and offers an innovative solution to the growing threat of bacterial resistance.

## 4 Experimental Section

### 4.1 Materials

The alginic acid sodium salt of medium viscosity (ALG), chitosan of medium molecular weight (CHIT), polyethylene imine (PEI) (molecular weight (Mw) of 600–1000 kDa), Span 80, Tween 80, thymol (purity >98,5%) (THY), menthol (MEN) and D_2_O (99.9% at D) were purchased from Sigma-Aldrich (Poznań, Poland). Poly­(acrylic acid) (*M*_w_ = 100 kDa) (PAA) and carvacrol (CAR) were obtained from Pol-Aura (Zabrze, Polska). *N*,*N*′-dicyclohexylcarbodiimide (DCC) and 4-dimethylaminopyridine (DMAP) were synthetic grade and purchased from Acros Organics (Geel, Belgium). Resveratrol (RES) of purity >98% was obtained from Linegal Chemicals. Acetic acid was analytical grade and purchased from PPH’ STANLAB’ Sp. J. (Lublin, Poland). Calcium chloride was obtained from Eurochem BGD. Sp. z o.o. (Tarnów, Polska). Dimethyl sulfoxide (DMSO), hexane, and acetone (all chemicals of analytical grade) were purchased from Avantor Performance Materials (Gliwice, Poland).

### 4.2 Synthesis of the PAA Decorated by THY, MEN or CAR

PAA modified with THY, MEN, or CAR was synthesized using Steglich esterification under mild conditions for the preparation of PEs functionalized with antimicrobial groups. Briefly: 41.7 mmol of carboxylic acid groups in PAA, THY (12.5 mmol for 5% substitution or 29.2 mmol for 15% substitution) or MEN (12.5 mmol for 5% substitution or 29.2 mmol for 15% substitution) or CAR (12.5 mmol for 5% substitution or 29.2 mmol for 15% substitution), a proper amount of DCC (16.3 or 37.7 mmol for 5% or 15% substitution, respectively) and a catalytic amount of DMAP were dissolved in 100–200 mL of DMSO. The molar ratio of carboxylic acid groups in PAA to essential oil (THY, MEN or CAR) to DCC was 1:0.3:0.4 for 5% substitution and 1:0.7:0.9 for 15% substitution. The mixture was stirred for 48 h at 22 °C. After that time, 2 mL of distilled water was dropped into the mixture to decompose unreacted DCC. The reaction mixture was stirred for an additional 2 h and then was filtered to remove the precipitated byproduct—dicyclohexylurea (DCU). After filtration, the obtained solution was dialyzed with distilled water (4 × 5L, 3 days, MWCO = 3500 Da). The resulting mixture was filtered, and the product was isolated by lyophilization.

### 4.3 ^1^H NMR Analysis

The new functionalized PAA derivatives were characterized by ^1^H NMR spectroscopy. Before analysis, samples were prepared by dissolving new PEs in D_2_O. The experiments were performed using the Bruker AMX500 instrument (Bruker, Billerica, MA, USA) at 25 °C. In ^1^H NMR spectra, chemical shifts refer to the deuterium hydrogen oxide (HDO) signal (4.71 ppm), as an internal standard with a spectral resolution of at least 0.730 Hz.

### 4.4 FTIR Spectroscopic Analysis

Fourier transform infrared (FTIR) spectra of new functionalized PAA derivatives as well as decorated nano- and microparticles were recorded with a spectrophotometer (IR Spirit, Shimadzu Corporation, Tokyo, Japan) equipped with a diamond crystal attenuated total reflection (ATR) accessory. Spectra were recorded in the range of 4000–400 cm^–1^, with a resolution of 2 cm^–1^ and 64 scans.

### 4.5 Fabrication of Hydrogel Microparticles with PE Coatings

Hydrogel microparticles comprising an ALG core were synthesized via an emulsion-based methodology.^11^ Initially, a 1.5% (w/v) ALG aqueous solution containing 1% (w/v) Tween 80 was added to hexane (1:4, v/v) containing 1% (w/v) Span 80, and the resulting mixture was emulsified in a round-bottom reactor using a mechanical stirrer (1000 rpm, 10 min) to obtain an emulsion. Next, a 0.6 M calcium chloride aqueous solution was homogenized with hexane (2:3, v/v) containing 1% (w/v) Span 80 and added dropwise to the aforementioned emulsion. Then, the prepared mixture was stirred for 60 min at 1000 rpm. Following the cross-linking process, the resulting ALG-based microparticles were extracted from the emulsion by the addition of acetone, rinsed with distilled water, and dried. The formation of microparticles with PE coatings was achieved through the LbL technique, utilizing CHIT as the polycation and PAA or PAA-X-DS% (X = THY, MEN, CAR; DS = 5, 15) as the polyanion. The initial step involved dispersing the microspheres in a 0.4% (w/v) CHIT aqueous solution of 2% acetic acid and stirring for 20 min to facilitate the adsorption of the first PE layer. Subsequently, the microparticles were dispersed in a 0.1% aqueous solution of PAA or PAA-X-DS% and gently stirred for 20 min to deposit the second PE coating. Following each PE adsorption, the microparticles were filtered and rinsed with distilled water. The obtained microparticles were then dried and stored at room temperature.

The preparation of RES-loaded microparticles was initiated with the dispersion of RES in an aqueous solution of alginate (ALG) at a concentration of 1.5% (w/v) and a weight ratio of 2:3 (w/w). That was accomplished through homogenization at a speed of 6000 rpm for 3 min. The remaining steps of the fabrication process were conducted using the same method as for the preparation of empty microparticles.

### 4.6 Fabrication of Hydrogel Nanoparticles with LbL Coatings

Hydrogel nanoparticles comprising an ALG core were synthesized via high-pressure homogenization (HPH).^30^ In the initial stage of the process, a 0.25% (w/v) ALG aqueous solution containing 1% (w/v) Tween 80 was emulsified with hexane (1:4, v/v) containing 1% (w/v) Span 80 using a mechanical stirrer (1000 rpm, 5 min). The pre-emulsion was then subjected to high-pressure homogenization at 500 bar for a total of five cycles. The homogenization process was conducted at room temperature. The subsequent stage was the cross-linking process. The emulsion obtained during the HPH process was transferred to a glass vial and 0.1 M calcium chloride aqueous solution, which had been homogenized with hexane (1:4, v/v) containing 1% (w/v) Span 80, was added during magnetic stirring. The emulsion was agitated for 24 h at room temperature in the presence of the cross-linking agent. Next, the hexane was removed from the resulting mixture, and the nanoparticles were isolated from the emulsion by the addition of acetone and centrifugation (10,000 rpm, 30 min). The supernatant was discarded, and the gel-like pellet was purified with acetone and distilled water by further centrifugation (10,000 rpm, 30 min). The resulting nanoparticle suspension was filtered through a syringe filter with a pore size of 0.45 μm. The entire procedure was repeated several times as part of the method development. In order to create LbL coatings, the nanoparticle surface was subjected to further modification through the adsorption of PE, utilizing the saturation method as part of the LbL approach.^4^ The PE solutions of CHIT (1 mg/mL) were employed as the polycation, whereas PAA or PAA-X-DS% (X = THY, MEN, CAR; DS = 5, 15) (5 mg/mL) were used as the polyanion, with the objective of constructing the outer layers of nanoparticles. In the formation of CHIT-coated nanoparticles, the suspension of negatively charged nanogels was mixed with varying volumes of polycation solution. The required amount of CHIT was selected empirically, with the nanoparticles’ zeta potentials monitored to determine the optimal outcome. The optimal zeta potential was achieved at the maximal point, after which it remained constant. The process of polyanion deposition was performed following the same procedure. Each PE adsorption was confirmed by the zeta potential measurements.

To prepare RES-loaded hydrogel nanoparticles, RES was initially dispersed in an aqueous solution of 0.25% (w/v) ALG (2:3, w/w) using homogenization (6000 rpm, 3 min). The remaining steps were conducted in a manner analogous to the procedure employed to prepare empty nanoparticles, as described above.

### 4.7 Particle Morphology, Size and Zeta Potential

The morphology of the nano- and microparticles was evaluated using a scanning electron microscope (SEM) (Tesla BS 300) operated at 10 kV. Furthermore, a transmission electron microscope (TEM) (FEI Tecnai G2 20 X-TWIN) was utilized to examine the resulting nanoparticles. The nanoparticles were also studied by atomic force microscope (NX10 Park System, Suwon, South Korea) with NSC14 or NCHR tip and scanning speed between 0.1 and 0.2 Hz operating in noncontact mode. Prior to the analysis, samples were diluted with double-distilled water to an appropriate concentration (0.1% or 0.05% by weight), allowed to adsorb on a cover glass surface, and left in a dry place at room temperature.

The size distribution and zeta potential of the hydrogel nanoparticles were determined by dynamic light scattering (DLS) using the Zetasizer Nano ZS (Malvern Instruments, UK). The samples were measured at 25 °C. Prior to undertaking DLS measurements, all samples were diluted with distilled water. Each measurement was performed a minimum of three times.

The sizes of the microparticles under investigation were examined using a polarizing microscope (Eclipse TE2000S, Nikon, Tokyo, Japan). The mean diameter (MD) of the size of microparticles was defined as the mean of the diameters of 100 randomly selected particles. The polydispersity index (PdI) of the size distribution was determined using the following equation

$$PdI={\left(\frac{SD}{MD}\right)}^{2}$$

where: SD is the standard deviation of the microparticles’ diameter and MD is their mean diameter.^11^

### 4.8 Encapsulation Efficiency and Loading Capacity

The encapsulation efficiency (EE) and loading capacity (LC) of RES loaded in hydrogel nano- and microparticles were determined by UV–vis spectroscopy using a Hitachi U-2900 spectrophotometer. Absorption spectra were recorded in the 200–1000 nm wavelength range at a scanning speed of 800 nm/min. Prior to the measurement, the obtained microcarriers were suspended in an ethanol–water solution (25:1, v/v) and stirred for 48 h at ambient temperature. To characterize the nanocarriers, the nanoparticles in an aqueous solution were diluted with ethanol in a 1:100 ratio (v/v). The amount of RES encapsulated in the nano- and microparticles was estimated spectrophotometrically at 307 nm using a previously prepared calibration curve. The encapsulation efficiency was calculated in accordance with the following equation

$$EE=\frac{m_{e}}{m_{i}}\times 100\%$$

where *m*_e_ is the mass of RES encapsulated in carriers, and *m*_i_ is the initial mass of RES used for encapsulation.

The loading capacity was determined by the following equation

$$EE=\frac{m_{e}}{m_{p}}\times 100\%$$

where *m*_p_ is the mass of obtained particles.

### 4.9 Drug Release Study

The release of RES from functionalized hydrogel nano- and microparticles was evaluated in phosphate buffer saline (PBS) solution (pH 7.4) at 37 °C. Initially, the RES-loaded carriers were suspended in PBS, placed into a dialysis bag with a molecular weight cutoff (MWCO) of 3500 Da, and immersed in a glass vial containing buffer solution. Then, the particles were incubated at 37 °C with stirring at a speed of 100 rpm. At designated time points, 0.3 mL samples were collected from the medium and replaced with an equal volume of fresh PBS. The amount of released RES was determined by measuring the absorbance at λ = 307 nm using a UV–vis spectrophotometer (Hitachi U-2900). All experiments were repeated twice. The data were subjected to analysis using the Korsmeyer–Peppas (KP) model.

### 4.10 QCM-D Analysis

The adsorption behavior of functionalized PAAs was investigated utilizing a quartz crystal microbalance with dissipation monitoring (QSense Biolin Scientific, Gothenburg, Sweden), equipped with piezoelectric quartz crystals covered with gold electrodes. Prior to the commencement of the experiments, the crystals were subjected to an ultrasonication process (30 min) in a 2% Hellmanex III solution (Hellma, Müllheim, Germany), followed by a drying phase with air. QCM-D enables the measurement of both the normalized resonant frequency (Δ*f*) and energy dissipation (Δ*D*) shifts. The gold-plated electrodes were excited at their fundamental frequency (4.95 MHz) and at the third, fifth, seventh, ninth, and 11th overtones. All measurements were conducted at room temperature with a constant flow rate of 0.150 mL/min. The experiments were initiated with distilled water to establish a baseline. The adsorption measurements were conducted by employing an alternating deposition of oppositely charged polyelectrolytes (PEs) on the crystals. The sensors were initially coated with a positively charged polyethylene imine (PEI), an anchor layer. This was achieved by introducing a 0.05% w/v PEI solution to the QCM-D cells. Subsequently, ALG (0.15%, w/v) was adsorbed and cross-linked with calcium ions in order to imitate the particle core. Then, CHIT (0.04%, w/v) and PAA-X-DS% (X = THY, MEN, CAR; DS = 5, 15) (0.05%, w/v) were deposited on the substrates in order to imitate the particle coating. Each PE adsorption step was followed by a washing step, in which distilled water was used to remove any nonadsorbed molecules. Throughout the entire process, the frequency and dissipation were recorded simultaneously as a function of time. The physicochemical parameters of the resulting coatings were estimated utilizing the S1 Smartfit or B1 Broadfit models and QSense Dfind Software. The mass of the rigid layers was determined based on the Sauerbrey equation, with a value of Δ*D* < 1 × 10^–6^ per 10 Hz.

$$\Delta m=-C\frac{\Delta f}{n}$$

where *C* is the crystal constant that equals 17.7 ng/cm^2^ Hz; *n* is the overtone number.

### 4.11 Spectroscopic Ellipsometry Analysis

The thickness of the dry PE coatings was analyzed using spectroscopic ellipsometry. Prior to commencing the measurements, the silicon wafers were subjected to a cleansing process involving the use of the piranha solution, followed by boiling four times in distilled water and drying with air. The wafers were then dip-coated with PEI (0.05%, w/v) as the initial layer, after which ALG (0.15%, w/v) was adsorbed on the top, followed by cross-linking with calcium ions. Subsequently, CHIT (0.04%, w/v) and PAA-X-DS% (X = THY, MEN, CAR; DS = 5, 15) (0.05%, w/v) were deposited. For each PE adsorption, the silicon wafers were immersed in the PE solution for 20 min, after which they were washed with distilled water. The prepared samples were finally stream-dried with air. The thickness and optical parameters of the coatings were determined as described in detail elsewhere.^31^

### 4.12 Antimicrobial Activity

The antimicrobial activities of new functionalized PAAs, as well as nano- and microparticles decorated by such PEs, were investigated against the Gram-positive bacterial strain (S. aureus PCM 2054) and the Gram-negative strain (E. coli PCM 2057). The strains were subcultured from the original culture and maintained in Nutrient LAB-AGAR TM (Biomaxima) plates at 4 °C at the Department of Organic and Medicinal Chemistry at Wroclaw University of Science and Technology.

#### 4.12.1 Agar Disc-Diffusion Assay

The bacterial strains were cultivated in Nutrient Broth (Biomaxima) for 24 h, after which the bacterial suspension was adjusted to 0.5 McFarland Standard. Before utilization, the Petri dishes containing Mueller–Hinton agar (Biocorpo) were allowed to dry for 15 min. Sterile filter paper discs (ø = 6 mm) were impregnated with a 10 μL solution of PEs/nanoparticles/microparticles suspension in ethanol (*C* = 10 mg/mL) and placed on agar plates with an inoculum of bacteria. Ethanol was selected as a solvent to ensure proper dispersion of the studied materials and uniform application onto the agar surface, due to its volatility and compatibility with a wide range of compounds. Although ethanol possesses inherent bactericidal properties, it was primarily used to control particles’ properties and therefore served as a negative control in this study. To facilitate diffusion, the plates were permitted to rest for 15 min at ambient temperature, then incubated at 37 °C for 24 h. Following this period, the inhibition zones formed around the discs were measured. The experiments were conducted in triplicate.

#### 4.12.2 Minimal Inhibitory Activity Evaluation

The bacterial strains were subcultured in Tryptone Soya Broth (Oxoid) and incubated at 37 °C for 18–24 h. After that, the turbidity of the inoculum was adjusted to obtain concentrations of 5 × 10^5^ CFU/mL (OD550 = 0.125). The MEN, THY and CAR solutions were diluted in DMSO (stock solution) and subsequently applied to the wells in the first column, with 164 μL of broth at a volume of 16 μL. The PEs and nanoparticles were suspended in broth and subsequently applied to the first column at a volume of 180 μL. The broth was added to columns 2–10 at a volume of 90 μL, while column 11 received 82 μL and column 12 received 82 μL. Serial double dilutions were prepared horizontally across the 96-well plate (NEST), spanning columns 1 to 9. The initial concentrations of MEN, THY and CAR were 3.20 mg/mL, while the starting concentrations of PEs and nanoparticles were 5 mg/mL. Excess dilutions (90 μL) were discarded from column 9. A volume of 10 μL of liquid culture was added to each well. The prepared plates were incubated for a period of 24 h at a temperature of 37 °C. Following the incubation period, 10 μL of alamarBlue reagent was added to columns 1–12, and the plates were further incubated at the appropriate temperature for 3 h before analysis. Column 10 (medium + inoculum) represented cell viability, while column 11 (medium + DMSO + inoculum) represented the lack of inhibitory effect of DMSO toward the microorganism (in the case of MEN, THY and CAR, the DMSO was replaced with broth). Column 12 (gentamycin at a concentration of 16 × 10^–3^ mg/mL + medium + inoculum) represented inhibited microorganism growth. The minimum inhibitory concentration (MIC) was defined as the lowest concentration of the test agent that caused significant growth inhibition. A change in the color of the alamarBlue dye was taken to indicate the viability of the microorganism, as described by Baker et al.^32^ The MIC was determined as the concentration of the test agent that remained blue.

## Supplementary Material



## Notes

Figure S1. FTIR spectra of the obtained functionalised polyelectrolytes (PAA-THY-5%, PAA-THY-15%, PAA-MEN-5%, PAA-MEN-15%, PAA-CAR-5% and PAA-CAR-15%). Figure S2. FTIR spectra of hydrogel microparticles functionalised with antimicrobial coatings. Figure S3. FTIR spectra of hydrogel nanoparticles functionalised with antimicrobial coatings (PDF)

## References

[1] Duan S., Wu R., Xiong Y., Ren H., Lei C., Zhao Y., Zhang X., Xu F. (2022). Multifunctional antimicrobial materials:
From rational design to biomedical applications. Prog. Mater. Sci..

[2] Ding X., Wang A., Tong W., Xu F. (2019). Biodegradable Antibacterial
Polymeric Nanosystems: A New Hope to Cope with Multidrug-Resistant
Bacteria. Small.

[3] Bahrami A., Delshadi R., Jafari S.-M. (2020). Active
delivery of antimicrobial
nanoparticles into microbial cells through surface functionalisation
strategies. Trends Food Sci. Technol..

[4] Kurapati R., Groth T.-W., Raichur A. M. (2019). Recent
Developments in Layer-by-Layer
Technique for Drug Delivery Applications. ACS
Appl. Bio Mater..

[5] Ghiorghita C.-A., Bucatariu F., Dragan E.-S. (2019). Influence of crosslinking in loading/release
applications of polyelectrolyte multilayer assemblies. A review. Mater. Sci. Eng., C.

[6] Sarode A., Annapragada A., Guo J., Mitragotri S. (2020). Layered self-assemblies
for controlled drug delivery: A translational overview. Biomaterials.

[7] Kenawy E., Worley S.-D., Broughton R. (2007). The Chemistry
and Applications of
Antimicrobial Polymers: A State-of-the-Art Review. Biomacromolecules.

[8] Belbekhouche S., Bousserrhine N., Alphonse V., Carbonnier B. (2019). From beta-cyclodextrin
polyelectrolyte to layer-by-layer self-assembly microcapsules: From
inhibition of bacterial growth to bactericidal effect. Food Hydrocolloids.

[9] Budincic J.-M., Petrovic L., D̵ekic L., Fraj J., Bucko S., Katona J., Spasojevic L. (2021). Study of Vitamin
E Microencapsulation
and Controlled Release from Chitosan/Sodium Lauryl Ether Sulfate Microcapsules. Carbohydr. Polym..

[10] Stan D., Enciu A., Mateescu A.-L., Ion A. C.-A., Brezeanu A.-C., Stan D., Tanase C. (2021). Natural Compounds
With Antimicrobial
and Antiviral Effect and Nanocarriers Used for Their Transportation. Front. Pharmacol.

[11] Szczęsna W., Tsirigotis-Maniecka M., Lamch Ł., Szyk-Warszyńska L., Zboińska E., Warszyński P., Wilk K.-A. (2022). Multilayered Curcumin-Loaded
Hydrogel Microcarriers with Antimicrobial Function. Molecules.

[12] Padilla N., Delso I., Bergua F., Lafuente C., Artal M. (2024). Characterization
of camphor: thymol or dl-menthol eutectic mixtures; Structure, thermophysical
properties, and lidocaine solubility. J. Mol.
Liq..

[13] Cengiz H.-Y., Konyali E., Müftüler A., Deligöz H. (2023). Investigating
the effect of weak polyelectrolytes on the chemical stability and
swelling recovery of multilayered coatings. Prog. Org. Coat..

[14] Wang Y., Zheng X., Wang Z., Shi Z., Kong Z., Zhong M., Xue J., Zhang Y. (2021). Effects of
–COOH
and –NH2 on adsorptive polysaccharide fouling under varying
pH conditions: Contributing factors and underlying mechanisms. J. Membr. Sci..

[15] Szczęsna-Górniak W., Weżgowiec J., Tsirigotis-Maniecka M., Szyk-Warszyńska L., Michna A., Warszyński P., Saczko J., Wilk K.-A. (2024). Physicochemical
Features and Applicability of Newly Fabricated Phytopharmaceutical-Loaded
Hydrogel Alginate Microcarriers with Viscoelastic Polyelectrolyte
Coatings. ChemPhysChem.

[16] Zhang W., He Q., Jiang S., Wang L. (2019). Comparative Analysis of Menthol,
Thymol, and Carvacrol for Antimicrobial Efficacy and Chemical Composition. J. Essent. Oil Res..

[17] Silhavy T.-J., Kahne D., Walker S. (2010). The bacterial cell envelope, Cold
Spring Harb. Perspect. Biol..

[18] Ait-Ouazzou A., Espina L., Gelaw T.-K., de Lamo-Castellví S., Pagán R., García-Gonzalo D. (2013). New Insights in Mechanisms
of Bacterial Inactivation by Carvacrol. J. Appl.
Microbiol..

[19] Trombetta D., Castelli F., Sarpietro M. G., Venuti V., Cristani M., Daniele C., Saija A., Mazzanti G., Bisignano G. (2005). Mechanisms
of Antibacterial Action of Three Monoterpenes. Antimicrob. Agents Chemother..

[20] Xu J., Zhou F., Ji B.-P., Pei R.-S., Xu N. (2008). The Antibacterial
Mechanism of Carvacrol and Thymol against Escherichia Coli. Lett. Appl. Microbiol..

[21] Lopez-Romero J.C., González-Ríos H., Borges A., Simões M. (2015). Antibacterial
Effects and Mode of Action of Selected Essential Oils Components against
Escherichia Coli and Staphylococcus Aureus. Evidence-Based Complementary Altern. Med..

[22] Gallucci M.-N., Oliva M., Casero C., Dambolena J., Luna A., Zygadlo J., Demo M. (2009). Antimicrobial Combined
Action of Terpenes against the Food-Borne Microorganisms Escherichia
Coli, Staphylococcus Aureus and Bacillus Cereus. Flavour Fragrance J..

[23] El
Atki Y., Aouam I., El Kamari F., Taroq A., Gourch A., Lyoussi B., Abdellaoui A. (2019). Antibacterial Efficacy of Thymol,
Carvacrol, Eugenol and Menthol as Alternative Agents to Control the
Growth of Nosocomial Infection-Bacteria. J.
Pharm. Sci. Res..

[24] Ben
Arfa A., Combes S., Preziosi-Belloy L., Gontard N., Chalier P. (2006). Antimicrobial
Activity of Carvacrol Related to Its Chemical Structure. Lett. Appl. Microbiol..

[25] Tsirigotis-Maniecka M., Szyk-Warszyńska L., Maniecki Ł., Szczęsna W., Warszyński P., Wilk K.-A. (2021). Tailoring the Composition of Hydrogel
Particles for the Controlled Delivery of Phytopharmaceuticals. Eur. Polym. J..

[26] Tsirigotis-Maniecka M., Szyk-Warszynska L., Lamch Ł., Wezgowiec J., Warszynski P., Wilk K.-A. (2021). Benefits of pH-responsive polyelectrolyte
coatings for carboxymethyl cellulose-based microparticles in the controlled
release of esculin. Mater. Sci. Eng., C.

[27] Hadadian M., Allahyari R., Mahdavi B., Mohammadhosseini M. (2024). Comparative
study of β-cyclodextrin and carboxymethyl-β-cyclodextrin
as effective drug delivery systems for oxymetholone: Design, preparation,
characterisation, phase solubility and in vitro drug release studies. J. Sci. Adv. Mater. Devices.

[28] Rungpetchanan T., Krathumkhet N., Sirivat A., Paradee N. (2023). Electrically controlled
transdermal drug release of ionic and non-ionic drug from kappa-iota
carrageenan cryogel. Mater. Chem. Phys..

[29] Ouedraogo S., Grosjean M., Brigaud I., Carneiro K., Luchnikov V., Mathieu N., Garric X., Nottelet B., Anselme K., Pieuchot L., Ponche A. (2024). Fabrication
and characterisation
of thin self-rolling film for anti-inflammatory drug delivery. Colloids Surf., B.

[30] Riegger B.-R., Kowalski R., Hilfert L., Tovar G.-E.-M., Bach M. (2018). Chitosan nanoparticles via high-pressure
homogenization-assisted miniemulsion crosslinking for mixed-matrix
membrane adsorbers. Carbohydr. Polym..

[31] Tsirigotis-Maniecka M., Szyk-Warszyńska L., Michna A., Warszyński P., Wilk K.-A. (2018). Colloidal Characteristics and Functionality of Rationally
Designed Esculin-Loaded Hydrogel Microcapsules. J. Colloid Interface Sci..

[32] Baker C.-N., Banerjee S.-N., Tenover F.-C. (1994). Evaluation
of Alamar Colorimetric
MIC Method for Antimicrobial Susceptibility Testing of Gram-Negative
Bacteria. J. Clin. Microbiol..

