# Supplementary material for: Novel Antimicrobial-Decorated Polyelectrolytes as Versatile Building Blocks for Multifunctional Hydrogel Nano- and Microparticles
Source: ACS Omega. 2025 May 20;10(21):22165–83. doi: 10.1021/acsomega.5c02518 (PMC12138636; doi:10.1021/acsomega.5c02518)
Supplement: Supplementary file 1 [file ao5c02518_si_001.pdf]

Electronic Supplementary Material (ESI)

Novel Antimicrobial-Decorated Polyelectrolytes as Versatile Building Blocks for Multifunctional Hydrogel Nano- and Microparticles

Weronika Szczęsna-Górniak[a,*], Łukasz Lamch[a], Alicja Surowiak[b], Ewa Zboińska[c], Lilianna Szyk-Warszyńska[d], Marcin Bartman[a], Piotr Warszyński[d], Kazimiera A. Wilk[a]

[a] Department of Engineering and Technology of Chemical Processes, Faculty of Chemistry, Wrocław University of Science and Technology, 50-370 Wrocław, Poland
[b] Department of Bioorganic Chemistry Faculty of Chemistry, Wrocław University of Science and Technology, 50-370 Wrocław, Poland
[c] Department of Organic and Medicinal Chemistry, Faculty of Chemistry, Wrocław University of Science and Technology, 50-370 Wrocław, Poland
[d] Jerzy Haber Institute of Catalysis and Surface Chemistry, Polish Academy of Sciences, 30-239 Kraków, Poland

Table of Contents

Figure S1. FTIR spectra of the obtained functionalised polyelectrolytes (PAA-THY-5%, PAA-THY-15%, PAA-MEN-5%, PAA-MEN-15%, PAA-CAR-5% and PAA-CAR-15%).

Figure S2. FTIR spectra of hydrogel microparticles functionalised with antimicrobial coatings.

Figure S3. FTIR spectra of hydrogel nanoparticles functionalised with antimicrobial coatings.

The FTIR spectra of the functionalised polyelectrolytes (PEs) revealed characteristic absorption bands confirming the incorporation of essential oils into the poly(acrylic acid) (PAA) backbone, as shown in Figure S1. Broad bands around 3400 cm^{-1} correspond to O–H stretching vibrations, which originate from the PAA polymer [1]. Peaks observed at approximately 2920 cm^{-1} and 2850 cm^{-1} are assigned to asymmetric and symmetric C–H stretching vibrations of aliphatic $–CH_2$ groups [1]. Intense absorption bands in the 1700–1600 cm^{-1} region are attributed to C=O stretching of the carboxylic acid groups present in PAA [1]. Shifts in this region and changes in band intensity suggest intermolecular interactions, such as hydrogen bonding, appearing upon functionalisation of the PAA with the essential oils [2,3]. Notably, spectral differences between the PEs decorated with thymol and carvacrol (PAA_THY and PAA_CAR) versus those modified with menthol (PAA_MEN) can be attributed to structural distinctions between the compounds [4]. Thymol and carvacrol are aromatic monoterpenoids featuring a phenolic ring, which contributes characteristic aromatic C=C stretching vibrations typically observed around 1600–1450 cm^{-1} [3,4]. These bands are clearly visible in the studied spectra and are more pronounced in the PAA grafted with a higher degree of functionalisation with thymol and carvacrol (15%) [3]. In contrast, menthol lacks an aromatic ring and instead features a saturated cyclohexane ring, leading to the absence of the aromatic-related peaks [4]. Instead, the menthol-modified PAA displays enhanced aliphatic C–H stretching and bending vibrations, consistent with its alicyclic structure [4,5]. Additionally, absorption bands in the 1200–1000 cm^{-1} range, corresponding to C–O stretching, are present in all spectra and may result from the esterification process [1,5]. The difference in shape and intensity of these peaks can potentially reflect various chemical environments and bonding characteristics introduced by each bioactive compound [2,3]. The observed spectral variations between the 5% and 15% of functionalisation support concentration-dependent interactions, as higher modification degree leads to more pronounced spectral changes due to the increased presence of functional groups from the essential oils [2,3].

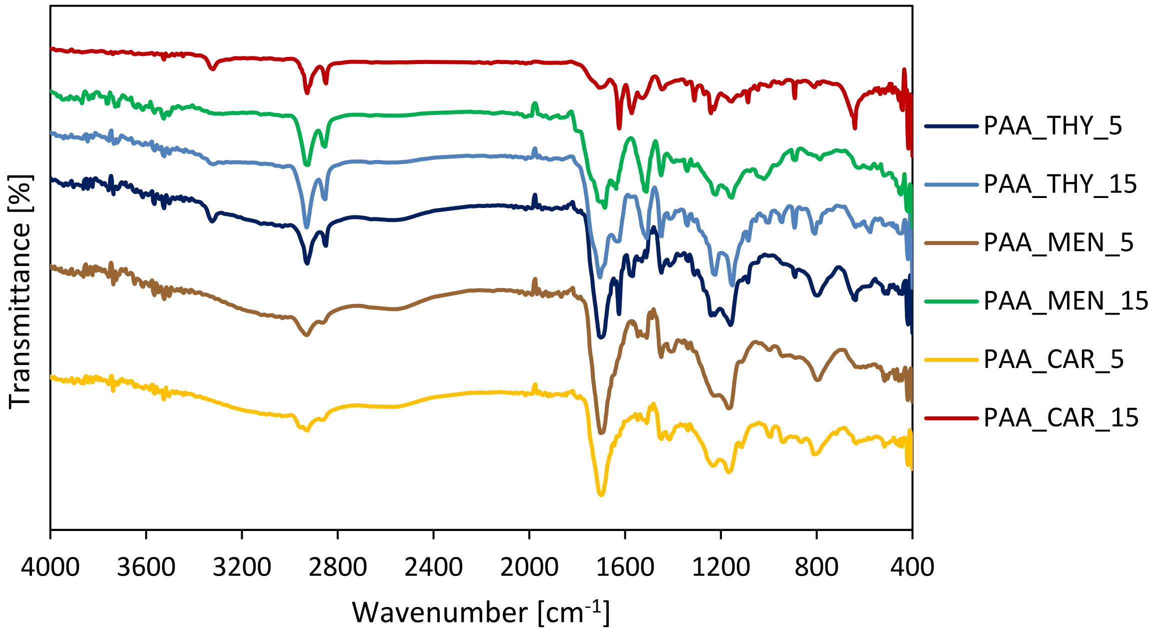

Figure S1. FTIR spectra of the obtained functionalised PEs (PAA-THY-5%, PAA-THY-15%, PAA-MEN-5%, PAA-MEN-15%, PAA-CAR-5% and PAA-CAR-15%).

The FTIR spectra of the functionalised microparticles (MT5, MT15, MM5, MM15, MC5, MC15), illustrated n Figure S2, exhibit the characteristic features of functionalized PAA, thus confirming the successful assembly of PE layers composed of PAA modified with thymol, menthol, or carvacrol onto a chitosan–coated alginate particle core, as presented in Figure S2. Broad bands around 3400 cm^{-1} correspond to O–H vibrations, reflecting the hydroxyl groups present in alginate, chitosan, and PAA [1,5]. The bands at approximately 2920 cm^{-1} and 2850 cm^{-1} are attributed to C–H stretching vibrations of aliphatic chains, particularly noticeable in microparticles decorated with menthol-modified PAA (MM5, MM15), which is consistent with the saturated cyclohexane ring of menthol [4]. A key feature is the strong absorption band between 1650–1600 cm^{-1}, corresponding to overlapping C=O stretching of carboxylic groups (from alginate and PAA) and N–H bending vibrations from chitosan [1,5]. The position and intensity of these peaks vary among the studied samples, indicating interactions between the PEs and the essential oils [2,3]. Microparticles decorated with thymol-grafted PAA (MT5, MT15) and carvacrol-grafted PAA (MC5, MC15), exhibit additional bands in the 1600–1450 cm^{-1} region due to aromatic C=C stretching vibrations of thymol and carvacrol aromatic phenolic rings [3,4]. These bands are absent in the particles coated with menthol-grafted PAA (MM5, MM15), in agreement with non-aromatic structure of menthol [4,5]. Moreover, the peaks in the 1200–1000 cm^{-1} range, attributed to C–O–C and C–O stretching vibrations, are influenced by both alginate and functionalised PAA ester/ether linkages, and show distinct profiles depending on the incorporated bioactive compound and its concentration [1,5]. The observed differences between particles decorated with 5% essential oil-modified PAA and those decorated with 15% of modification indicate a concentration-dependent deposition of the functionalized PAA layer, which is reflected by increased band intensity in microsystem coatings with higher functionalization degree [2,3].

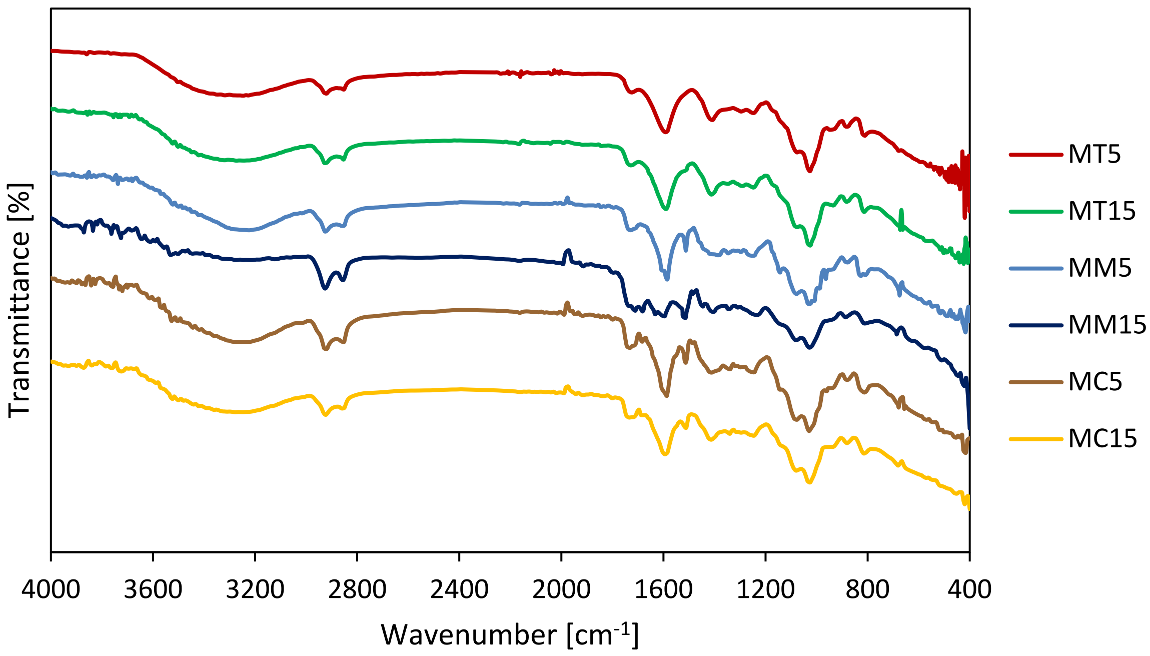

Figure S2. FTIR spectra of hydrogel microparticles functionalised with antimicrobial coatings.

The FTIR spectra of the decorated nanoparticles (NT5, NT15, NM5, NM15, NC5, NC15) confirm the effective adsorption of PE coatings on the particle core and are shown in Figure S3. As for microparticles, all spectra exhibited characteristic bands associated with the nanosystem components. Broad absorption bands around 3200–3400 cm^{-1} correspond to O–H and N–H stretching vibrations, indicative of hydroxyl and amine groups present in alginate and chitosan, respectively [1,5]. The presence of asymmetric and symmetric stretching of carboxylate groups (COO^{-}) was confirmed by peaks near ~1600 cm^{-1} and ~1400 cm^{-1}, typically attributed to alginate and PAA [1]. The peaks observed around ~2960–2860 cm^{-1} can be ascribed to C–H stretching of methyl groups of bioactive substance [3,4]. All spectra showed bands in the region of ~1100 cm^{-1} associated with C–O stretching, confirming the adsorption of essential oils-grafted PAA layers [1,5]. The presence of aromatic C=C stretching vibrations around ~1600 cm^{-1} supports the successful deposition of thymol-modified PAA and carvacrol-modified PAA onto chitosan-coated nanoparticles [3,4]. Additionally, bands in the 800–850 cm^{-1} range correspond to aromatic ring deformations, characteristic of thymol and carvacrol [4]. Overall, shifts in peak positions and intensity variations across the spectra suggest various interactions, including hydrogen bonding and hydrophobic interaction, between PEs layers, confirming successful nanoparticles modification using different functional coatings [2,3].

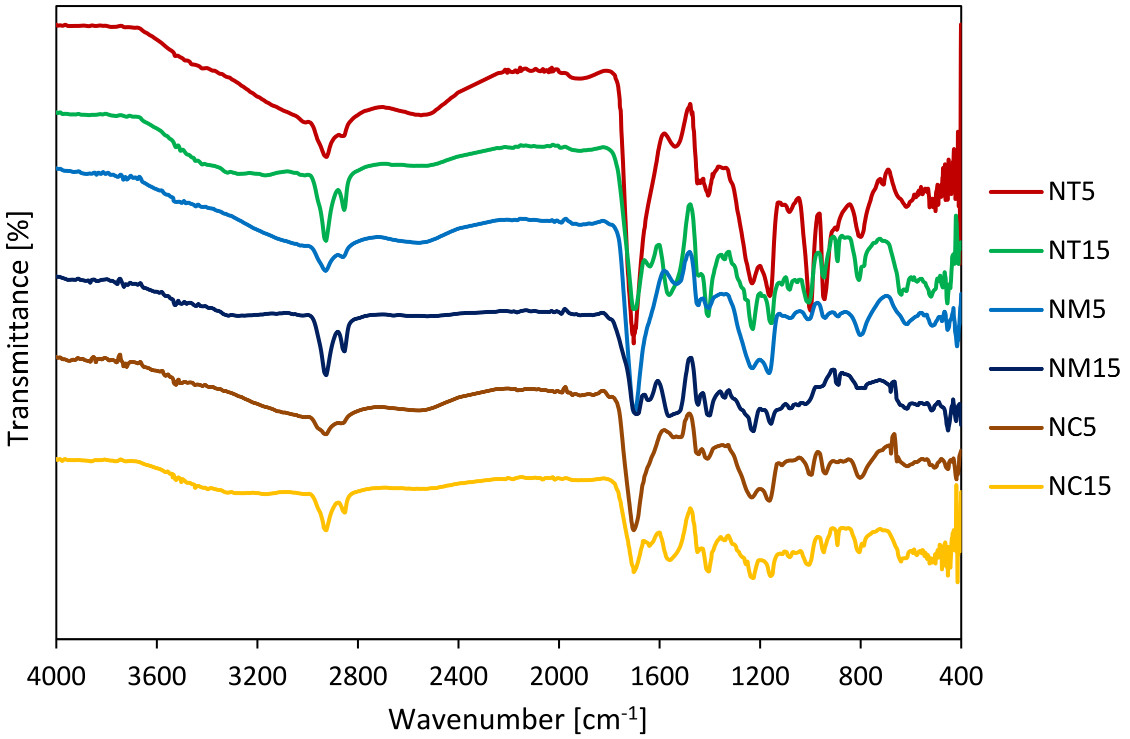

Figure S3. FTIR spectra of hydrogel nanoparticles functionalised with antimicrobial coatings.

References:

1. Mbah CJ, Builders PF, Agubata CO, Attama AA. Fourier Transform Infrared Spectroscopy for Functional Group Analysis of Polymers. *J Appl Polym Sci.* 2018;135(31):46571. doi:10.1002/app.46571
2. Mansouri S, Lavigne P, Bérubé M, Hildgen P. Encapsulation of Essential Oils into Nanoparticles: Characterization and Release Kinetics. *Colloids Surf B Biointerfaces.* 2015;136:1100–1106. doi:10.1016/j.colsurfb.2015.10.050
3. Marchese A, Orhan IE, Daglia M, et al. Antimicrobial and Antioxidant Activities of Thymol, Carvacrol and Related Isoprenoids. *Nat Prod Res.* 2017;31(5):597–605. doi:10.1080/14786419.2016.1219108
4. Trombetta D, Castelli F, Sarpietro MG, et al. Mechanisms of Antibacterial Action of Three Monoterpenes (Thymol, Carvacrol, and Menthol) on Staphylococcus aureus. *Planta Med.* 2005;71(12):1189–1194. doi:10.1055/s-2005-873009
5. Singh A, Narvi SS, Dutta PK, Pandey ND. Chitosan: A Promising Biopolymer for Site-Specific Drug Delivery. *Int J Biol Macromol.* 2011;48(4):540–545. doi:10.1016/j.ijbiomac.2011.01.020
